# Let the Model Choose Its Own Frequency: An Adaptive Frequency-Aware Inverted Transformer for Noise-Robust Gearbox Fault Diagnosis

**DOI:** 10.3390/s26144622

**Published:** 2026-07-21

**Authors:** Sohaib Arshad Mayo, Hafiz Tayyab Mustafa, Mujtaba Asad, Hamza Mustafa, Saud Rehman, Zhiqiang Cai

**Affiliations:** 1 School of Mechanical Engineering, Northwestern Polytechnical University, Xi’an 710072, China; sohaibarshad@mail.nwpu.edu.cn (S.A.M.);; 2 School of Computer Science and Technology, Zhejiang Normal University, Jinhua 321004, China; mustafa.tayyab@zjnu.edu.cn; 3 School of Automation & Intelligent Sensing, Shanghai Jiao Tong University, Shanghai 200240, China; asadmujtaba@sjtu.edu.cn; 4 Department of Electrical and Information Engineering, University of Cassino and Southern Lazio, 03043 Cassino, Italy

**Keywords:** gearbox fault diagnosis, adaptive frequency filtering, iTransformer, residual attention, FiLM conditioning, noise robustness, cross-channel attention

## Abstract

Gearbox fault diagnosis under noisy operating conditions remains a critical yet unsolved challenge for industrial condition monitoring. The primary challenge originates from a fundamental conflict: fault signatures are present within certain frequency ranges, yet standard deep learning models process raw vibration signals without recognizing which frequencies are relevant. Moreover, noise affects the entire spectrum uniformly. Existing transformer-based methods for vibration analysis treat time steps as tokens and therefore fail to capture cross-sensor dependencies, while conventional denoising approaches apply fixed filters that cannot adapt to the varying spectral characteristics of different fault types and noise levels. We propose the Adaptive Frequency-Aware Inverted Transformer (AF-iTransformer), a lightweight transformer framework that lets the model learn which frequencies to attend to on a per-sample basis. In particular, we propose a learnable spectral filter that transforms the input signal to the frequency domain via FFT. Then it predicts a soft frequency mask conditioned on signal statistics and applies it before reconstructing the filtered signal through iFFT, allowing the model to suppress noise bands while preserving fault-relevant spectral content dynamically. After adaptive filtering, the architecture employs channel-level tokenization to uniformly represent heterogeneous channels as input tokens, relying on cross-channel attention to automatically learn their distinct contributions to fault diagnosis. Feature-wise linear modulation is introduced to inject signal-level statistics at every encoder layer. Furthermore, the framework utilizes residual attention propagation to stabilize deep training, and an auxiliary spectrum prediction head provides spectral regularization during training. On the UConn Gearbox dataset with nine fault categories, AF-iTransformer achieves 99.63% accuracy on clean data and maintains 95.1% at 0 dB signal-to-noise ratio, substantially outperforming all baselines under noisy conditions. On the SEU gearbox dataset with five fault categories, AF-iTransformer achieves 99.74% clean accuracy. On 2 GB edge GPUs, AF-iTransformer achieves a per-window inference latency of 7.3–13 ms with peak memory below 17 MB, and 1.4–4.7 ms on modern CPUs, confirming its viability for real-time industrial deployment.

## 1. Introduction

Gearboxes are critical components in wind turbines, helicopters, mining trucks, electric vehicles, and industrial drivetrains. Unplanned gearbox failure causes prolonged downtime and costly repairs [1,2]. The economic impact is substantial. Replacing a single wind turbine gearbox can cost hundreds of thousands of dollars. Unexpected downtime in mining and manufacturing leads to large revenue losses per hour [3]. These realities have driven intensive research into automated fault diagnosis systems that provide early warning of impending failures [4,5]. Vibration-based condition monitoring is the most widely adopted diagnostic approach. Mechanical faults produce characteristic acceleration signatures that can be captured by mounted sensors and analyzed automatically [1,2].

Traditional fault diagnosis relied on hand-crafted signal processing techniques. These include fast Fourier transform, envelope analysis, wavelet decomposition, and time-synchronous averaging [1,6]. This approach works well under controlled laboratory conditions but has two fundamental shortcomings. First, it requires domain expertise to select the right frequency bands for each fault type and each machine configuration, which does not scale across different gearbox designs. Second, and more critically, these fixed filters are helpless when the noise floor rises above the fault signature because they cannot adapt to what the signal should look like under the current conditions [3,7].

Deep learning (DL) enabled end-to-end fault classification by learning discriminative features from raw or minimally processed vibration data [4,5]. Convolutional neural network (CNN)-based methods such as WDCNN [8], multi-scale CNNs [9], and the deep residual shrinkage network [10] have been proposed to solve fault classification. However, CNN-based methods share a fundamental limitation for noise robustness. Convolutional filters respond to local patterns and process every feature without the ability to selectively filter the frequency content before processing. When those patterns are corrupted by noise, the feature maps become unreliable. Furthermore, most CNN architectures process multi-channel data through simple channel concatenation. This prevents the model from learning cross-channel noise-cancellation patterns [11,12].

Transformer-based models have recently been adapted for vibration diagnostics. They can capture long-range dependencies through self-attention [13,14]. Vision Transformers and their variants have been applied to vibration spectrograms [15,16]. Swin Transformer has been adapted for bearing diagnosis [17,18]. YConvFormer combines convolutional features with transformer attention for gearbox diagnosis [19]. However, these approaches treat the temporal axis as the token axis. They do not optimally capture cross-channel dependencies when multiple vibration channels are available.

The iTransformer [20] offers a promising alternative by inverting the conventional transformer tokenization: instead of treating time steps as tokens, it promotes each variate (sensor channel) to a token, which is a natural inductive bias for multi-channel fusion. Yet the iTransformer has never been adapted for vibration data with rotating machinery physics, and it lacks any mechanism for frequency-aware preprocessing or operating-condition adaptation. Furthermore, existing approaches handle domain shifts through separate domain-adaptation stages [21] rather than providing native conditioning within the model architecture, adding computational overhead without addressing the root cause of the problem: the spectral content of the signal itself changes under different operating conditions and noise levels.

Our key insight is that fault diagnosis under noisy conditions is fundamentally a frequency selection problem. The fault information lives in specific, predictable frequency bands, while noise spreads across the entire spectrum. If we can teach the model to identify and emphasize the right frequencies for each input sample, the downstream classification becomes far more robust. This is not the same as applying a fixed bandpass filter, because the optimal frequency selection depends on the current signal characteristics: a heavily ‘noised’ sample may need stronger suppression of high-frequency bands, while a clean sample benefits from preserving the full spectrum.

**Figure 1:**
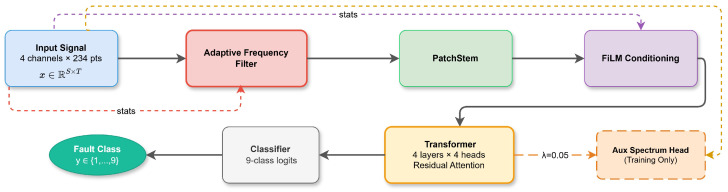
Overview of the proposed Adaptive Frequency-Aware Inverted Transformer for noise-robust gearbox fault diagnosis.

In this paper, we propose the Adaptive Frequency-Aware Inverted Transformer (AF-iTransformer), which embeds this insight directly into the architecture. The model learns which spectral components to pass and which to suppress for every input sample, utilizing a soft frequency mask. This mask is predicted from signal-level statistics (RMS amplitude, peak-to-peak range, spectral centroid) through a lightweight MLP, so it adapts to the noise level and fault characteristics of each individual sample. A residual gate ensures that the original signal is never fully discarded, preserving information that the mask might accidentally suppress. After adaptive filtering, channel-level tokenization projects each channel into a single embedding, so that cross-channel attention becomes the primary computation. A transformer encoder with feature-wise linear modulation (FiLM) conditioning and residual attention propagation processes these tokens for fault classification, while an auxiliary spectrum prediction head provides additional supervision by requiring the encoder to preserve frequency-domain information. The overview of the proposed framework is presented in Figure 1. Our main contributions are as follows.

- We introduce a learnable adaptive frequency filter that operates in the spectral domain (FFT, predicted mask, iFFT) with a dual-branch mask combining sample-specific and global components, plus a residual gate that prevents information loss.
- We propose channel-level tokenization, where each channel becomes a token, enabling cross-channel attention as the primary computation. This design naturally captures how fault energy propagates between sensor or channel locations.
- We integrate FiLM conditioning from signal-derived statistics at every encoder layer, enabling the model to adapt its processing to the current signal characteristics without separate domain-adaptation stages.
- We combine residual attention propagation and an auxiliary spectrum head that together stabilize training and provide spectral regularization.
- We demonstrate that AF-iTransformer achieves 99.63% clean accuracy on the UConn Gearbox 9-class dataset and 99.74% on the SEU planetary gearbox 5-class dataset, and we validate noise robustness under three representative noise types (AWGN, impulsive, and harmonic interference) on both datasets, confirming that the adaptive frequency filtering principle is dataset-agnostic.

The remainder of this paper is organized as follows. Section 2 reviews related work. Section 3 presents the architecture in detail. Section 4 describes the experimental setup. Section 5 reports results on clean and noisy data. Section 6 presents ablation studies. Section 7 discusses findings, limitations, and future work. Section 8 concludes the paper.

## 2. Related Work

### 2.1. Deep Learning for Vibration-Based Fault Diagnosis

The shift from hand-crafted features to end-to-end deep learning (DL) has transformed vibration-based diagnostics over the past decade. Zhang et al. [8] introduced WDCNN with wide first-layer kernels that serve as learnable bandpass filters, showing that deep networks could extract discriminative features from raw vibration signals without manual feature engineering. Huang et al. [9] utilized parallel convolutional streams with different kernel sizes to capture features at multiple scales.

The deep residual shrinkage network [10] incorporated soft thresholding as a learnable nonlinearity to suppress noise. Shang et al. [22] embedded differentiable wavelet denoising as network layers. Ni et al. [23] incorporated bearing kinematic physics as inductive bias into a ResNet. Despite these advances, CNN-based methods share common limitations: they process multi-channel data through channel concatenation rather than modeling inter-channel dependencies, and their convolutional filters operate in the time domain without explicit frequency selection, which limits their noise robustness when the signal-to-noise ratio drops below 0 dB [3].

### 2.2. Transformer-Based Fault Diagnosis

Recently, transformer-based models have been increasingly applied to fault diagnostics due to their ability to capture long-range dependencies through self-attention [13,14]. The Vision Transformer (ViT) and its variants process vibration data by converting signals to 2D representations such as Gramian Angular Field images [15] and time-frequency spectrograms [16].

Zhou et al. [15] combined GAF images with a CNN-ViT hybrid. The Swin Transformer has been adapted for bearing diagnosis under variable conditions [17,24,25], and Cao et al. [18] proposed ARAM-Swin with adaptive residual attention for variable-speed diagnosis. However, all these methods treat the temporal axis as the token axis, which does not naturally support cross-channel attention when multiple sensors are available. The iTransformer [20] inverts this by promoting each variate to a token, which is ideal for multi-channel fusion, yet it has not been specialized for vibration data and lacks frequency-aware preprocessing or operating-condition conditioning. Our work fills this gap by equipping the iTransformer paradigm with adaptive spectral filtering and signal-conditioned modulation.

### 2.3. Noise-Robust Fault Diagnosis

Noise robustness has become a critical concern in vibration-based fault diagnosis. Researchers increasingly recognize that high clean-data accuracy does not guarantee reliable deployment in industrial environments. Zhang et al. [26] proposed cross-attention deep residual shrinkage networks for gearbox fault diagnosis in noisy environments. Their method combines noise suppression with cross-attention mechanisms. Liu et al. [27] introduced a lightweight multi-scale depthwise separable convolution for gearbox diagnosis under high noise. Ma et al. [28] combined multiscale depthwise separable convolution with bidirectional GRU and squeeze-and-excitation attention for gearbox diagnosis under noisy conditions. Yang et al. [29] proposed a reliability-guided adaptive feature fusion network for noise-robust bearing fault diagnosis. Their network dynamically adjusts feature fusion weights based on estimated feature reliability under noisy conditions. Hu et al. [30] introduced an interpretable shapelet-based model for noise-robust bearing diagnosis in CNC machine tools. Xu et al. [31] proposed an attention-driven multi-scale framework for rotating machinery fault diagnosis under noisy conditions. However, these methods address noise through preprocessing denoising or CNN modifications. None of them leverage the cross-channel attention mechanism that is central to our approach, and none employ the iTransformer channel-as-token paradigm.

### 2.4. Multi-Channel Fusion and Feature Modulation

Multi-sensor fusion for intelligent fault diagnosis has been introduced by Kibrete et al. [11] and Lin et al. [12]. Fusion strategies range from naive channel concatenation to attention-based approaches: Cao et al. [32] proposed a wavelet-enhanced CNN-transformer hybrid with cross-attention, and Wang et al. [33] fused multi-sensor data via efficient channel attention. For handling operating-condition variability, domain adaptation techniques [34,35] have been widely studied but require separate training stages or adversarial discriminators. Feature-wise linear modulation (FiLM) [36], originally developed for visual reasoning, has been applied to fault diagnosis by Zhang et al. [37] for cross-condition CNN-based diagnosis. However, FiLM has not been used within a transformer encoder for fault diagnosis, and existing fusion approaches do not model cross-channel dependencies through attention. Our channel-as-token formulation makes cross-channel attention the primary computation, and we integrate it with adaptive filtering and FiLM conditioning within a unified architecture.

### 2.5. Learnable Spectral Filtering for Signal Enhancement

Spectral filtering is a cornerstone of vibration signal processing, but it has traditionally been applied as a fixed preprocessing step using manually designed bandpass filters or wavelet denoising [1,6]. Recent work has explored making spectral filtering learnable: Shang et al. [22] embedded differentiable wavelet denoising with learnable thresholds within a CNN, and the deep residual shrinkage network [10] uses soft thresholding as a learnable nonlinearity. However, these approaches operate on time-domain features after convolution rather than directly in the frequency domain. Frequency-domain learning has been explored in audio processing and speech enhancement [38], where learnable masks are applied to the STFT representation, but this idea has not been applied to vibration-based fault diagnosis. Our adaptive frequency filter brings learnable spectral masking to vibration diagnostics, operating directly on the FFT representation with a dual-branch mask design that balances sample-specific adaptation with global stability.

## 3. Methodology

### 3.1. Problem Formulation

Let $x\in R^{S\times T}$ denote vibration signals from *S* accelerometer channels, each containing *T* time samples. The goal is to map *x* to a fault class y∈{1,2,…,C} while being robust to additive noise *n* at varying signal-to-noise ratios (SNRs). In practice, the observed signal is $\tilde{x}=x+n$, and the model must learn to extract fault-discriminative features despite the corruption. This formulation explicitly recognizes that noise robustness is not merely a desirable property but a core requirement for real-world deployment, where sensors capture vibration in environments with electromagnetic interference, structural resonance, and operating-condition transients that contaminate the signal across the entire frequency spectrum. All modules are parameterized by *S* and *T*: the adaptive frequency filter’s mask prediction network adjusts its input dimension to 3S and output dimension to S×F, where F=T/2+1, and channel-level tokenization produces N=S tokens. The architecture therefore scales naturally from S=1 (UConn, single released accelerometer axis, T=3600) to S=4 (SEU, four distributed vibration sensors, T=1024) without architectural modification.

### 3.2. Architecture Overview

**Figure 2:**
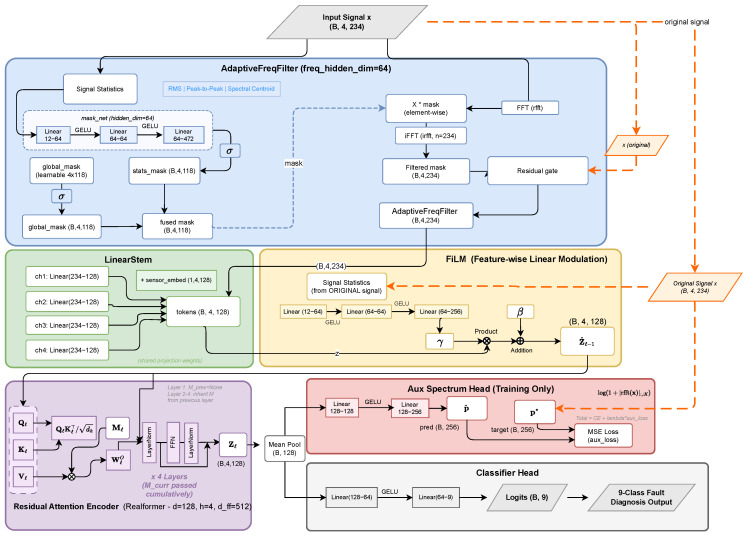
Overall architecture of AF-iTransformer. Multi-channel vibration signals $x\in R^{S\times T}$ are first processed by the AdaptiveFreqFilter (FFT → mask prediction → iFFT with residual gate), then tokenized by the Stem module into S×P tokens, and processed by an *L*-layer inverted transformer encoder with FiLM conditioning and residual attention propagation. A two-layer MLP classification head predicts fault classes, while an auxiliary spectrum head (dashed, training only) provides spectral regularization.

Figure 2 shows the end-to-end architecture of AF-iTransformer. The framework processes multi-channel vibration signals through five integrated modules that operate in sequence. First, the Adaptive Frequency Filter (AdaptiveFreqFilter) transforms the input signal to the frequency domain via FFT, predicts a soft frequency mask conditioned on signal statistics, applies element-wise masking, and reconstructs the filtered signal via iFFT, with a residual gate that fuses the filtered signal with the original to prevent information loss. Second, the channel-level tokenization module (LinearStem) projects each sensor channel’s filtered signal to the embedding dimension via a linear layer, producing *S* tokens with sensor identity encoding, following the iTransformer paradigm where variables become tokens. Third, a Transformer encoder with FiLM conditioning processes the token sequence through *L* layers of self-attention where cross-channel dependencies are modeled, with FiLM signal-conditioned modulation applied before each attention computation. Fourth, the Auxiliary Spectrum Prediction Head regularizes the encoder during training by requiring it to preserve frequency-domain information that can reconstruct the original signal’s spectrum. Fifth, the Classification Head aggregates tokens via mean pooling and produces fault-class logits through a two-layer MLP.

The key design principle is that frequency-first processing: rather than letting the transformer discover which frequencies matter from corrupted time-domain data, we explicitly teach the model to filter the spectrum before tokenization. This shifts the burden of noise suppression from the attention mechanism, which must simultaneously learn noise patterns, fault patterns, and their interactions, to a dedicated spectral filter that handles noise at the source.

### 3.3. Adaptive Frequency Filter

Fault signatures in gearbox vibration occupy specific frequency bands, including gear-mesh harmonics, bearing characteristic frequencies, and their sidebands, while additive white Gaussian noise spreads uniformly across the entire spectrum. A model that processes the raw time-domain signal must learn to separate fault content from noise using only its internal representations, which becomes increasingly difficult as the SNR drops. We argue that frequency-domain filtering should happen before the transformer processes the signal, and critically, the filter should be adaptive: it should learn which frequencies to pass for each individual sample based on its characteristics, rather than applying a fixed filter to all samples.

#### 3.3.1. Physical Motivation for Frequency-Selective Filtering

Gearbox vibration signals have a well-defined spectral structure governed by the kinematics of rotating components. For a gearbox with *Z* teeth on the gear and shaft frequency $f_{s}$, the gear mesh frequency (GMF) is given by the following:

(1) $$f_{\mathrm{mesh}}=Z\times f_{s}$$

Local faults such as tooth cracks, missing teeth, and spalling produce amplitude and phase modulation of the gear mesh harmonics, generating sidebands spaced at the shaft rotation frequency $f_{s}$ around the mesh frequency and its harmonics. The sideband structure encodes fault type and severity: a missing tooth produces strong, evenly spaced sidebands; root cracks generate weaker but still detectable sidebands; surface faults produce sidebands with characteristic asymmetry depending on the loading zone [1].

On the UConn dataset ($f_{s}\approx 22.22$ Hz output-shaft frequency after the 30 Hz input reduction, $Z_{1}=32$ teeth on stage 1), the primary gear-mesh frequency is ${\mathrm{GMF}}_{1}=711.11$ Hz with harmonics at 1422.22 Hz and 2133.33 Hz, and fault-related sidebands appear at ${\mathrm{GMF}}_{1}\pm n\;\cdot \;f_{s}$ for n=1,2,… On the SEU planetary gearbox dataset ($f_{s}\approx 20$ Hz motor frequency), the planetary GMF is approximately 320 Hz and the ring-planet mesh frequency is approximately 280 Hz, each with its own sideband families. Critically, this spectral structure means that fault information is concentrated in specific, predictable frequency bands (the mesh harmonics and their sidebands), while additive noise spreads uniformly across the entire spectrum. This physical separation between signal and noise in the frequency domain is the foundational motivation for the adaptive frequency filter: rather than processing the entire spectrum equally, the filter learns to preserve the narrow bands carrying fault information while suppressing the broadband noise between them. Table 1 summarizes the per-class dominant spectral signatures on the UConn dataset, including dominant spectral content and physical correspondence.

#### 3.3.2. Dual-Branch Frequency Mask

The adaptive frequency filter operates in three stages: (1) transform the input to the frequency domain, (2) predict a soft frequency mask, and (3) apply the mask and reconstruct via inverse FFT. The key design is a dual-branch mask that combines a sample-specific branch (which adapts to each input) with a global branch (which provides a stable baseline):

Step 1: Signal Statistics Computation. For each channel *s*, we compute three statistics from the time-domain input that capture the signal’s operating condition and noise level:

(2) $$q\left[s\right]=\left(\mathrm{RMS}(x[s]),\;P2P(x[s]),\;\mathrm{Cent}(x[s])\right)\in R^{3}$$

where $\mathrm{RMS}\left(x\left[s\right]\right)=\sqrt{\frac{1}{T}\sum_{t}x{\left[s,t\right]}^{2}}$ measures the signal energy, $P2P\left(x\left[s\right]\right)=\max_{t}x\left[s,t\right]-\min_{t}x\left[s,t\right]$ captures the dynamic range, and $\mathrm{Cent}\left(x\left[s\right]\right)=\frac{\sum_{k}f_{k}\left|X\left[s,k\right]\right|}{\sum_{k}\left|X\left[s,k\right]\right|}$ is the spectral centroid computed from the magnitude FFT |X[s,k]| at frequency bin $f_{k}$. The per-channel statistics are concatenated: $q=\left[q\left[1\right];\ldots ;q\left[S\right]\right]\in R^{3S}$.

Step 2: Sample-Specific Mask Prediction. A three-layer MLP predicts a per-channel frequency mask from the statistics:

(3) $$m_{\mathrm{stats}}=\sigma \;\left(\mathrm{MaskNet}(q)\right)\in R^{S\times F}$$

where F=T/2+1 is the number of frequency bins from the real FFT, σ denotes the sigmoid function, and MaskNet is a three-layer MLP (hidden dimension $H_{f}=64$, GELU activation) that maps $R^{3S}\to R^{S\times F}$. The output layer of MaskNet is initialized with zero weights and bias b=2.0, so that σ(2.0)≈0.88 at initialization and the mask starts near pass-through.

Step 3: Global Learnable Mask. A channel-specific global mask $m_{\mathrm{global}}\in R^{S\times F}$ is parameterized as a learnable tensor initialized to 2.0 (yielding σ(2.0)≈0.88). This branch does not depend on the input sample and provides a stable baseline mask that captures frequency patterns common across all samples.

Step 4: Dual-Branch Blending. The two mask branches are blended with a learnable gate α:

(4) $$m=\sigma \left(\alpha \right)⊙m_{\mathrm{stats}}+\left(1-\sigma \left(\alpha \right)\right)⊙m_{\mathrm{global}}$$

where α is initialized to 0.5 on UConn and 0.75 on SEU (giving σ(0.5)≈0.62 and σ(0.75)≈0.68, both slightly favoring the sample-specific branch). The dual-branch design balances adaptation and stability: the sample-specific branch adjusts the mask for each input’s noise level and fault type, while the global branch provides a stable filter that does not degrade when input statistics are unreliable under heavy noise. The initialization α=0.5 provides a gradient signal to MaskNet from the first batch while keeping the global branch active during early training; full justification and the empirical α sweep experiment are reported in Section 6.2.  

Step 5: Frequency-Domain Filtering. The input signal is transformed to the frequency domain, the mask is applied element-wise, and the filtered signal is reconstructed:

(5) $$\begin{array}{c} X=\mathrm{rfft}\left(x\right)\in C^{S\times F} \end{array}$$

(6) $$\begin{array}{c} X_{\mathrm{filt}}=X⊙m \end{array}$$

(7) $$\begin{array}{c} x_{\mathrm{filt}}=\mathrm{irfft}\left(X_{\mathrm{filt}},n=T\right)\in R^{S\times T} \end{array}$$

Step 6: Residual Gate. A learnable gate controls the blending of filtered and original signals:

(8) $$x_{\mathrm{out}}=\sigma \left(g\right)⊙x_{\mathrm{filt}}+\left(1-\sigma \left(g\right)\right)⊙x$$

where *g* is a scalar parameter initialized to −2.0 (giving σ(−2.0)≈0.12), so the model initially passes mostly the original signal and gradually learns to rely on the filtered version as training progresses. This residual connection is critical for noise robustness: it ensures that the original signal is never fully discarded, preventing the mask from accidentally removing fault-relevant content.

The adaptive frequency filter offers several advantages that together make it a robust and practical module. The mask is predicted from input statistics, so it automatically adjusts to different noise levels and fault types without manual tuning. The global mask branch prevents degenerate behavior when input statistics are unreliable under heavy noise. The residual gate ensures that filtering never destroys the original signal, making the module safe to use even when the predicted mask is imperfect. The entire FFT, mask, and iFFT pipeline is end-to-end differentiable, allowing the filter to be optimized jointly with the rest of the architecture.

### 3.4. Channel-Level Tokenization (LinearStem)

Following the iTransformer [20], we adopt channel-level tokenization in which each sensor channel becomes a token. In the conventional transformer, time steps are tokens and variates are features; the iTransformer inverts this convention so that variates become tokens and time steps become the embedding dimension. For gearbox diagnostics, multiple accelerometers measure the same mechanical process from different locations, and the relationships between channels encode information about fault type, location, and severity. This channel-as-token paradigm makes cross-channel relationships the primary object of attention.

In the primary configuration, each channel’s filtered signal is projected to the embedding dimension through a single linear layer:

(9) $$z\left[s\right]=x_{\mathrm{out}}\left[s\right]\;W_{\mathrm{proj}}+b_{\mathrm{proj}}\in R^{D}$$

where $W_{\mathrm{proj}}\in R^{T\times D}$ is the projection matrix. This produces N=S tokens per sample, one per sensor channel. We add a learnable sensor identity encoding to each token:

(10) $$z_{0}\left[s\right]=z\left[s\right]+e_{\mathrm{sensor}}\left[s\right]$$

where $e_{\mathrm{sensor}}\in R^{S\times D}$ encodes which accelerometer the token came from. This encoding allows the attention mechanism to distinguish between channels and learn physically meaningful cross-sensor dependencies. The token matrix is $Z_{0}\in R^{N\times D}$. For the UConn configuration (S=1, T=3600, D=128), this yields N=1 token (the single released accelerometer axis); for SEU (S=4, T=1024, D=128), it yields N=4 tokens. The architecture adapts automatically to both configurations without modification.

With the LinearStem tokenization strategy paired with a wider adaptive frequency filter ($H_{f}=64$), each channel already contains clean discriminative information because the filter effectively suppresses noise before tokenization. The cross-channel attention over four tokens then captures how fault energy propagates between sensor locations, which is the most important relationship for classification.

This channel-as-token design resolves the ”confusion-by-design” problem of temporal transformers, where vibration and tachometer channels are fused within every time-step token before any explicit channel-level reasoning can occur. By making each channel a separate token, the attention matrix $A\in R^{S\times S}$ directly models pairwise channel relationships, allowing the model to learn that cross-sensor attention (SEU, 4 distributed sensors) should differ depending on the physical relationship between channels. On UConn (S=1), the attention matrix is trivially 1×1 and the model relies on the adaptive frequency filter and FiLM conditioning. The sensor identity encoding (Equation (10)) reinforces this by giving the attention mechanism an explicit signal about channel identity on multi-channel datasets. The tachometer signal in the UConn rig is used solely for TSA preprocessing and is not a model input token.

### 3.5. Feature-Wise Linear Modulation

A classifier trained under one noise level or operating condition may lose accuracy under another, even when the underlying fault state is unchanged [39]. Rather than treating this as a domain adaptation problem that requires separate training stages, we argue that the model should be able to adjust its internal representations based on the current signal characteristics, in much the same way that FiLM conditioning works in visual reasoning [36].

#### FiLM Mechanism

The FiLM module computes per-channel statistics from the original (unfiltered) input signal, not the filtered signal. Using the original signal ensures that the modulation parameters reflect the true noise level and signal characteristics before any frequency-domain processing alters them. The same statistics defined in Equation (2) are computed from *x* and used to produce affine modulation parameters:

(11) $$\left(\gamma ,\beta \right)={\mathrm{MLP}}_{\mathrm{FiLM}}\left(q\right)\in R^{2D}$$

where ${\mathrm{MLP}}_{\mathrm{FiLM}}$ is a three-layer MLP (hidden dimension $H_{f}$, GELU activation). At each transformer layer *ℓ*, the token representations are modulated before attention computation:

(12) $${\hat{Z}}_{\ell -1}=\left(1+\gamma \right)⊙Z_{\ell -1}+\beta$$

where ⊙ denotes element-wise multiplication with broadcasting across the *N* token dimension. The last layer of ${\mathrm{MLP}}_{\mathrm{FiLM}}$ is zero-initialized so that at the start of training γ=0 and β=0, giving 1+γ=1, which preserves the identity transformation. The same (γ,β) computed once from the input modulates all tokens at every encoder layer, providing a consistent signal-conditioned affine transformation that enables fine-grained adaptation without additional training stages.

Statistics are computed from the pre-filter (original) signal so that FiLM retains noise-level information the filter would otherwise suppress; the pre- vs. post-filter ablation is reported in Section 6.4.

### 3.6. Residual Attention Propagation

In a standard transformer, each layer computes attention independently:

(13) $$A_{\ell }=\mathrm{softmax}\;\left(Q_{\ell }K_{\ell }^{⊤}/\sqrt{d_{k}}\right)$$

The softmax operation can saturate attention weights early in deep transformers, making it difficult for subsequent layers to redistribute attention [40]. This is particularly problematic for vibration diagnostics, where the cross-channel attention patterns should be consistent across layers.

#### Accumulated Attention Logits

Following RealFormer [40], we accumulate the pre-softmax attention logits across layers:

(14) $$\begin{array}{c} M_{\ell }=M_{\ell -1}+\frac{Q_{\ell }K_{\ell }^{⊤}}{\sqrt{d_{k}}},\;M_{0}=0 \end{array}$$

(15) $$\begin{array}{c} A_{\ell }=\mathrm{softmax}(M_{\ell }) \end{array}$$

(16) $$\begin{array}{c} h_{\ell }=A_{\ell }V_{\ell } \end{array}$$

Each layer adds its attention logits to the running total, and softmax is applied to the accumulated logits at each layer. This means layer *ℓ* computes attention from the cumulative evidence of layers 1 through *ℓ*. In the gearbox setting, this allows each layer to build upon the cross-channel attention patterns established by preceding layers, which empirically stabilizes training and enables four attention layers without learning-rate warm-up.

The accumulation in Equation (14) applies only to the pre-softmax attention logits ($M_{\ell }$); the FFN retains the standard per-layer skip connection (Equations (21)–(23)). We do not accumulate FFN outputs across layers because their per-token nonlinear composition is already handled by the standard residuals, and accumulating them would blur per-layer specialization without the training-stability benefit observed for attention logits.

### 3.7. Transformer Encoder Layer

Each encoder layer combines FiLM-conditioned self-attention, residual connections, layer normalization, and a feed-forward network. FiLM modulation (Equation (12)) is applied once to $Z_{0}$ before the first encoder layer; subsequent layers receive the (already modulated) representation $Z_{\ell -1}$ from the previous layer’s residual output. The forward computation for layer *ℓ* consists of four main steps. First, the input token matrix is modulated by signal-conditioned parameters:

(17) $${\hat{Z}}_{\ell -1}=\left(1+\gamma \right)⊙Z_{\ell -1}+\beta$$

In the second step, the multi-head QKV projections are computed from the modulated input, and residual attention is applied:

(18) $$\begin{array}{c} Q_{\ell }={\hat{Z}}_{\ell -1}W_{\ell }^{Q},\;K_{\ell }={\hat{Z}}_{\ell -1}W_{\ell }^{K},\;V_{\ell }={\hat{Z}}_{\ell -1}W_{\ell }^{V} \end{array}$$

(19) $$\begin{array}{c} M_{\ell }=M_{\ell -1}+Q_{\ell }K_{\ell }^{⊤}/\sqrt{d_{k}} \end{array}$$

(20) $$\begin{array}{c} h_{\ell }=\mathrm{softmax}\left(M_{\ell }\right)\;V_{\ell }\;W_{\ell }^{O} \end{array}$$

where $W_{\ell }^{O}\in R^{D\times D}$ is the output projection matrix. Next, a residual connection and layer normalization (LN) are employed as follows:

(21) $$Z_{\ell }^{\prime }=\mathrm{LayerNorm}\;\left(h_{\ell }+Z_{\ell -1}\right)$$

The residual connection adds the unmodulated input $Z_{\ell -1}$, not the FiLM-modulated ${\hat{Z}}_{\ell -1}$. This ensures that the signal-conditioned modulation affects the attention computation but does not alter the residual gradient highway. Finally, the feedforward network (FFN) with a residual connection from the previous LN is utilized to produce final embeddings $Z_{L}\in R^{N\times D}$.

(22) $$\begin{array}{c} \mathrm{FFN}(x)=\mathrm{GELU}\left(xW_{1}+b_{1}\right)W_{2}+b_{2} \end{array}$$

(23) $$\begin{array}{c} Z_{\ell }=\mathrm{LayerNorm}\;\left(\mathrm{FFN}\left(Z_{\ell }^{\prime }\right)+Z_{\ell }^{\prime }\right) \end{array}$$

### 3.8. Auxiliary Spectrum Prediction Head

To encourage the encoder to learn representations that preserve frequency-domain information, rather than relying solely on time-domain shortcuts, we add a lightweight auxiliary head that predicts the spectral content of the original (unfiltered) signal from the encoder bottleneck. This serves as spectral regularization: the encoder must maintain enough frequency information in its representations to reconstruct the spectrum, which discourages it from discarding the very features that the adaptive filter has enhanced.

The auxiliary head predicts a *K*-bin log-magnitude spectrum from the pooled encoder output:

(24) $$\hat{p}=\mathrm{AuxHead}\;\left(\frac{1}{N}\sum_{n=1}^{N}Z_{L}\left[n\right]\right)\in R^{S\times K}$$

where AuxHead is a two-layer MLP (D→D→S×K) with GELU activation. The target is computed from the original input signal (not the filtered signal):

(25) $$p^{*}=\log\;\left(1+{\left|\mathrm{rfft}\left(x\right)\right|}_{:,:K}\right)\in R^{S\times K}$$

Using the original signal as the target is critical: if we used the filtered signal, the auxiliary head would simply learn to predict the mask-shaped spectrum, providing no useful regularization. The auxiliary loss is

(26) $$L_{\mathrm{aux}}=\frac{1}{SK}{∥\hat{p}-p^{*}∥}_{2}^{2}$$

The auxiliary head is only utilized during training of the network.

### 3.9. Classification Head

The final embedding is pooled across tokens to produce a global representation:

(27) $$z=\frac{1}{N}\sum_{n=1}^{N}Z_{L}\left[n\right]\in R^{D}$$

The classification head is a two-layer MLP with GELU activation and dropout:

(28) $$y={\mathrm{MLP}}_{\mathrm{cls}}\left(z\right)\in R^{C}$$

followed by softmax to obtain a probability distribution over *C* fault classes.

### 3.10. Loss Function

The total training loss combines classification cross-entropy, auxiliary spectrum regression, and mixup augmentation:

(29) $$L_{\mathrm{total}}=L_{\mathrm{CE}}+\lambda L_{\mathrm{aux}}$$

where λ=0.05 on UConn and λ=0.01 on SEU are the auxiliary loss weights, each selected based on the lambda sweep results provided in Section 6.1. During mixup augmentation (applied for the first 80% of training), the classification loss becomes:

(30) $$L_{\mathrm{CE}}^{\mathrm{mixup}}=\lambda_{m}L_{\mathrm{CE}}\left(y,y_{a}\right)+\left(1-\lambda_{m}\right)L_{\mathrm{CE}}\left(y,y_{b}\right)$$

where $\lambda_{m}∼\mathrm{Beta}\left(\alpha ,\alpha \right)$ with α=0.3, and $y_{a},y_{b}$ are the mixed labels. Label smoothing of 0.1 is applied to the cross-entropy loss. Algorithm 1 summarizes one forward pass through AF-iTransformer.
**Algorithm 1** AF-iTransformer Forward Pass.**Require:** Vibration $x\in R^{S\times T}$ **Ensure:** Class logits $y\in R^{C}$, auxiliary loss $L_{\mathrm{aux}}$  1:  q←ComputeStats(x){Equation (2)}   2:  $m_{\mathrm{stats}}\leftarrow \sigma \left(\mathrm{MaskNet}\left(q\right)\right)${Equation (3)}   3:  $m\leftarrow \sigma \left(\alpha \right)⊙m_{\mathrm{stats}}+\left(1-\sigma \left(\alpha \right)\right)⊙m_{\mathrm{global}}${Equation (4)}   4:  $x_{\mathrm{filt}}\leftarrow \mathrm{irfft}\left(\mathrm{rfft}\left(x\right)⊙m\right)${Equations (5)–(7)}   5:  $x_{\mathrm{out}}\leftarrow \sigma \left(g\right)⊙x_{\mathrm{filt}}+\left(1-\sigma \left(g\right)\right)⊙x${Equation (8)}   6:  $Z_{0}\leftarrow \mathrm{LinearStem}\left(x_{\mathrm{out}}\right)${Equations (9) and (10)}   7:  $\left(\gamma ,\beta \right)\leftarrow {\mathrm{MLP}}_{\mathrm{FiLM}}\left(q\right)${Equation (11)}   8:  $M_{0}\leftarrow 0$
  9:  **for**
ℓ=1,…,L
**do**
10:     ${\hat{Z}}_{\ell -1}\leftarrow \left(1+\gamma \right)⊙Z_{\ell -1}+\beta${Equation (17)} 11:     $Q_{\ell },K_{\ell },V_{\ell }\leftarrow {\hat{Z}}_{\ell -1}W_{\ell }^{Q,K,V}${Equation (18)} 12:     $M_{\ell }\leftarrow M_{\ell -1}+Q_{\ell }K_{\ell }^{⊤}/\sqrt{d_{k}}${Equation (19)} 13:     $h_{\ell }\leftarrow \mathrm{softmax}\left(M_{\ell }\right)V_{\ell }W_{\ell }^{O}${Equation (20)} 14:     $Z_{\ell }^{\prime }\leftarrow \mathrm{LayerNorm}\left(h_{\ell }+Z_{\ell -1}\right)${Equation (21)} 15:     $Z_{\ell }\leftarrow \mathrm{LayerNorm}\left(\mathrm{FFN}\left(Z_{\ell }^{\prime }\right)+Z_{\ell }^{\prime }\right)${Equation (23)}16:  **end for**
17:  $z\leftarrow \mathrm{MeanPool}(Z_{L})${Equation (27)} 18:  $y\leftarrow {\mathrm{MLP}}_{\mathrm{cls}}\left(z\right)${Equation (28)} 19:  $\hat{p}\leftarrow \mathrm{AuxHead}\left(z\right);\;p^{*}\leftarrow \log\left(1+|\mathrm{rfft}\left(x\right)|\right)${Equations (24) and (25)} 20:  $L_{\mathrm{aux}}\leftarrow \mathrm{MSE}\left(\hat{p},p^{*}\right)${Equation (26)} 21:  **return**
*y*, $L_{\mathrm{aux}}$


## 4. Experimental Setup

### 4.1. Datasets

UConn Dataset: The UConn Gearbox dataset [41,42] contains vibration signals from a 2-stage spur gearbox test rig in the Dynamic Systems & Control Laboratory at the University of Connecticut. The original experimental rig was instrumented with a 3-axis PCB accelerometer (X, Y, Z axes at one housing location) and a tachometer for shaft-speed measurement. The tachometer signal is used solely for time-synchronous averaging (TSA) preprocessing to align every record to the same angular position and is not provided to the model as an input. The publicly released dataset has a single TSA-aligned vibration signal per record (one axis of the triaxial accelerometer), sampled at $f_{s}=20$ kHz via a dSPACE DS1006 board. We therefore treat UConn as a single-channel dataset (S=1). The 936 records, each comprising T=3600 samples (0.18 s, corresponding to exactly 4 input-shaft revolutions at 900 samples/rev), are organized into 9 fault classes of 104 records each: Healthy (H), Missing Tooth (MT), Root Crack (RC), Surface Spalling (SP), and five Chipping severity levels (CH5 = most severe through CH1 = least severe). All faults are seeded on the Stage-1 32-tooth pinion. The gearbox comprises Stage 1 (32-tooth pinion meshing with an 80-tooth gear) and Stage 2 (48-tooth pinion meshing with a 64-tooth gear). The input-shaft frequency, verified by FFT analysis of the released records, is $f_{\mathrm{in}}=22.22$ Hz (1,333 RPM), yielding kinematic frequencies: Stage 1 GMF =32×22.22=711.11 Hz; Stage 1 output-shaft frequency =22.22×32/80=8.89 Hz; Stage 2 GMF =48×8.89=426.67 Hz; Stage 2 output-shaft frequency =8.89×48/64=6.67 Hz. Because all faults are on the Stage-1 pinion, fault-characteristic modulation appears as sidebands spaced at $f_{\mathrm{in}}=22.22$ Hz around the Stage-1 GMF (711.11 Hz).

SEU Gearbox Dataset: The Southeast University (SEU) gearbox dataset [43] contains vibration signals from a planetary gearbox test rig. The full release provides eight sensor channels (motor vibration, planetary X/Y/Z, motor torque, parallel X/Y/Z); we use the four standard planetary channels, motor vibration + planetary X/Y/Z, consistent with prior gearbox-diagnosis work, treating each sensor as one model input channel (S=4). Five health conditions are present: healthy, missing tooth, root crack, surface fault, and chipped tooth. Data are collected at 51.2 kHz under the 20 Hz/0 V operating condition. We use all available samples with a sample length of 1024 points, yielding approximately 5000 samples, split 70/15/15. The planetary gear mesh frequency is approximately 320 Hz, and the ring-planet mesh frequency is approximately 280 Hz.

These two datasets differ in several key dimensions such as number of channels (1 vs. 4), channel type (single released axis vs. distributed sensors), number of classes (9 vs. 5), signal length (3600 vs. 1024), sampling rate, and mechanical configuration (parallel-spur vs. planetary gearbox), making them complementary for validating the generality of AF-iTransformer. The UConn setting (single-channel) isolates the contribution of the adaptive frequency filter and FiLM conditioning without any cross-channel attention, while the SEU setting (four-channel) exercises the full cross-sensor attention mechanism across physically distributed sensors.

### 4.2. Dataset Visualization

**Figure 3:**
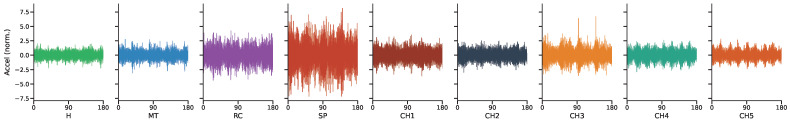
Representative clean vibration waveforms for the nine fault classes of the UConn Gearbox dataset (one window per class, 234 points at 64 kHz). Each class exhibits a characteristic temporal pattern: healthy signals are quasi-periodic at the mesh frequency, missing-tooth faults produce periodic high-amplitude impulses once per shaft revolution, root cracks and spalling introduce amplitude-modulated regimes, and the five chip-severity levels show progressively stronger impulsive content.

**Figure 4:**
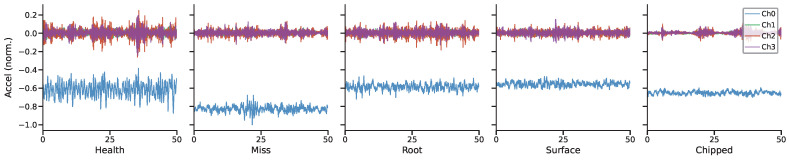
Representative clean vibration waveforms for the five fault classes of the SEU planetary gearbox dataset (one window per class, 1024 points at 51.2 kHz). Planetary-gearbox signals exhibit denser impulsive structure than the parallel-shaft UConn rig because of simultaneous sun–planet and ring–planet meshing. Missing-tooth and chipped faults produce strong periodic impulses at the planetary mesh frequency; surface and root faults produce more distributed modulation.

**Figure 5:**
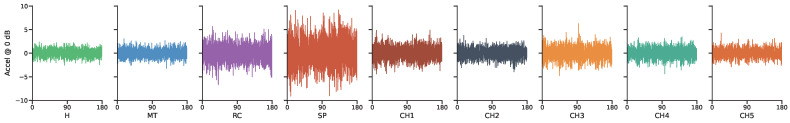
Same UConn waveforms as in Figure 3 after corruption by 0 dB AWGN (signal and noise equal power). The class-discriminative temporal structure visible in the clean waveforms is largely obliterated by eye, motivating the frequency-domain adaptive filter: while the time-domain signal appears noise-like, the spectral peaks at the gear-mesh frequency and its sidebands remain recoverable (see Figure 7).

**Figure 6:**
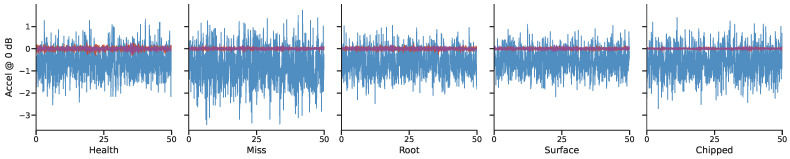
Same SEU waveforms as in Figure 4 after corruption by 0 dB AWGN. The planetary-mesh impulse trains that are clearly visible in the clean case are masked by broadband noise; only the largest impulses survive. This visualization motivates the cross-sensor attention mechanism: at 0 dB the model must combine information across the four SEU channels to recover the fault signature, since no single channel retains sufficient SNR.

**Figure 7:**
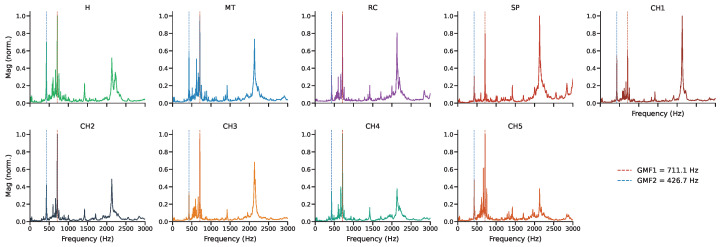
Per-class FFT magnitude spectra for the nine UConn fault classes (clean, 0–10 kHz range, log-scale y-axis). Vertical markers indicate the stage-1 gear-mesh frequency ${\mathrm{GMF}}_{1}\approx 711$ Hz and its first two harmonics (1422 Hz, 2133 Hz). Each fault type produces a distinct spectral signature: healthy and chip-1 classes show only the GMF peaks, missing-tooth produces strong sidebands spaced at the output-shaft frequency $f_{s}\approx 22.22$ Hz around ${\mathrm{GMF}}_{1}$, root crack produces fewer sidebands with growing amplitude with crack depth, and spalling excites broadband high-frequency content above ${\mathrm{2GMF}}_{1}$ (≈ 1422 Hz). These per-class spectral differences are the physical basis for the adaptive frequency mask.

To illustrate the signal characteristics of the two benchmarks used in this study, Figure 3, Figure 4, Figure 5, Figure 6 and Figure 7 present representative vibration waveforms and magnitude spectra for each fault class under both clean and 0 dB AWGN conditions. The UConn dataset (Figure 3, Figure 5 and Figure 7) spans nine health conditions: healthy, missing tooth, root crack, spalling, and five chip-severity levels, sampled at 64 kHz with 234 points per window. The SEU dataset (Figure 4 and Figure 6) covers five planetary-gearbox health conditions sampled at 51.2 kHz with 1024 points per window. Comparing the clean and 0 dB panels makes explicit the visual degradation that the adaptive frequency filter must compensate for: at 0 dB, the time-domain waveforms become indistinguishable from noise to the human eye, yet the per-class FFT spectra (Figure 7) retain weak but class-specific peaks at the gear-mesh frequency ($f_{m}\approx 450$ Hz for UConn) and its sidebands, which the filter learns to selectively preserve.

### 4.3. Implementation Details

All experiments are implemented in PyTorch 2.10.0 and trained on a single NVIDIA RTX 4090 GPU. We use the AdamW optimizer [44] with an initial learning rate of $2\times 10^{-3}$, a weight decay of $1\times 10^{-4}$, and a one-cycle cosine schedule over 200 epochs with a 15% warm-up fraction. The batch size is 128. Model hyperparameters include an embedding dimension of D=128, L=4 encoder layers, h=4 attention heads, a key dimension of $d_{k}=D/h=32$, an FFN expansion factor of 4, a FiLM hidden dimension of 64, an adaptive filter hidden dimension of 64, K=64 auxiliary spectrum bins, and auxiliary loss weights of λ=0.05 on UConn and λ=0.01 on SEU. Dropout of 0.1 is applied to attention weights and FFN outputs. Label smoothing of 0.1 is used in the cross-entropy loss. Gradient clipping at a max norm of 1.0 and early stopping with a patience of 50 epochs are used to prevent overfitting.

### 4.4. Data Augmentation Strategies

Our augmentation pipeline consists of two stages: a recording-session-level data split that prevents inter-sample leakage, and a multi-crop test-time augmentation that ensembles predictions over multiple views of the same input. We do not apply additive noise augmentation during training; the noise-robustness evaluation is therefore an out-of-distribution test of the trained model.

Recording-session-level split. The raw data recordings are first partitioned into train/validation/test splits at the recording-session level rather than at the window level. All overlapping-window crops derived from a given recording go into the same split, preventing leakage of recording-specific patterns (e.g., sensor mounting, ambient vibration floor) into the test set. This protocol is crucial for the UConn dataset, where overlapping-window augmentation is employed to expand the training set. If windows from the same recording were allowed to span multiple splits, the test accuracy would be optimistically biased by inter-sample correlation.

Train-time augmentation. On the training split only, we apply overlapping-window augmentation with window sizes [200, 170, 140] and a stride of 10, which expands the UConn training set from 2519 raw samples to approximately 55,418 windows. We also apply amplitude jitter (×0.97–1.03) throughout training to simulate the gain variability that arises from sensor mounting differences and pre-amplifier calibration drift. We do not inject additive Gaussian noise during training, because doing so would conflate the architectural contribution to noise robustness with the data-augmentation contribution.

Test-time augmentation. At evaluation, we employ multi-crop windowing with window sizes [234, 210, 190, 170, 150, 130] and strides [1, 10, 10, 10, 10, 10], combined with amplitude scaling factors [0.95, 1.0, 1.05] and temperature scaling at T=0.9. Predictions are averaged across all crops and scales. Multi-crop windowing captures the model’s predictions on multiple sub-windows of the same recording, which reduces variance from localized noise bursts and improves robustness to window-boundary effects. Test-time augmentation is applied identically to all methods (baselines and AF-iTransformer) to ensure a fair comparison.

Training–evaluation decoupling. All models, including AF-iTransformer and all baselines, are trained exclusively on clean data. No noise augmentation is applied during training. Noise is applied only at evaluation time to measure the trained model’s robustness to out-of-distribution signal corruptions. This protocol isolates the architectural contribution to noise robustness from any data-augmentation effect, ensuring a fair cross-architecture comparison.

## 5. Results

We organize the results into two parts: Part I evaluates AF-iTransformer on the UConn Gearbox 9-class dataset (the primary benchmark used in prior work), and Part II evaluates the same architecture on the SEU planetary gearbox dataset under two complementary sub-tasks—Experiment I (5-class gear-only classification, the focus of this paper) and Experiment II (9-class full-dataset evaluation combining gear and bearing faults). This two-dataset, three-experiment design demonstrates the generalization capability of the adaptive frequency filtering mechanism across different gearbox topologies, channel counts, and class granularities.

**Figure 8:**
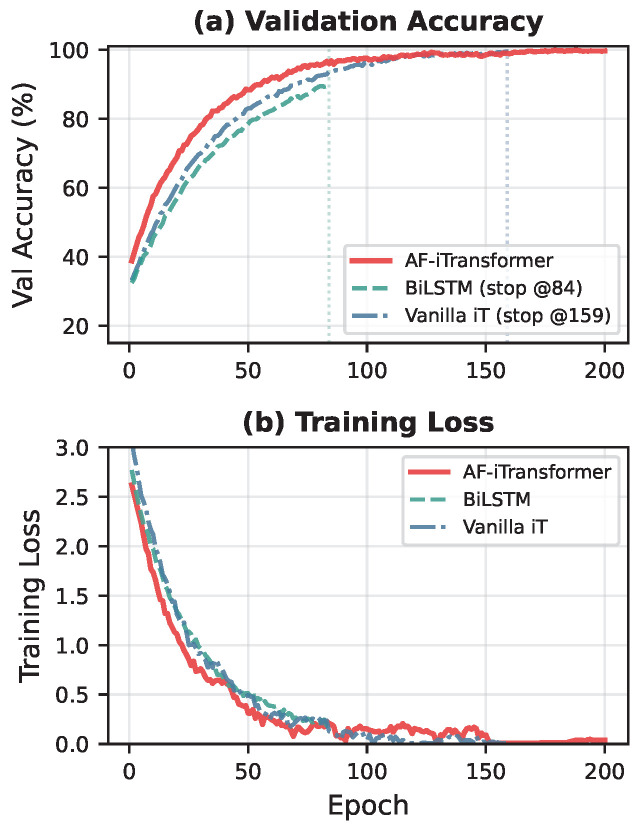
Training and validation curves for AF-iTransformer. (**a**) Cross-entropy loss. (**b**) Classification accuracy. The model converges rapidly with minimal overfitting.

Figure 8 shows the training and validation loss and accuracy curves for AF-iTransformer over 200 epochs. The model converges rapidly within the first 50 epochs, achieving training accuracy above 99%, and continues to refine its representations throughout the remaining epochs. The validation accuracy closely tracks the training accuracy with minimal overfitting, thanks to the combined effects of amplitude-jitter augmentation, label smoothing, dropout, and early stopping. The loss curve shows a smooth descent without oscillation, indicating stable optimization with the AdamW optimizer and cosine learning rate schedule.

### 5.1. Results on UConn Gearbox Dataset

Table 2 compares AF-iTransformer with baselines and published methods on the UConn Gearbox dataset under clean conditions. The vanilla iTransformer achieves 99.45%, confirming that the proposed method provides a strong baseline, while our adaptive frequency filter and other enhancements close the remaining gap and provide the critical advantage under noisy conditions.

The results highlight several observations. AF-iTransformer achieves the highest accuracy among all methods, including the vanilla iTransformer, which shares the same channel-level tokenization paradigm. This improvement comes from the adaptive frequency filter, which refines the input signal before the encoder processes it, even under clean conditions. The vanilla iTransformer and Transformer-1D both reach 99.45%, showing that the channel-as-token paradigm alone matches standard transformer performance on clean data. Traditional convolutional and recurrent methods (1D-CNN at 93.72%, BiLSTM at 98.71%) lag behind, confirming that attention-based models are better suited for fine-grained fault discrimination across multiple channels.

Among published methods on the UConn dataset, CBAM-ResNeXt50 [49] achieves 99.45%, matching our vanilla iTransformer baseline, while MSDARN [48] reaches 99.32% and Pro-MobileNetV3 [47] achieves 98.45%. These three methods were re-evaluated by Zhan et al. [41] using CEEMD preprocessing, which decomposes the signal into intrinsic mode functions before classification, whereas our method operates directly on raw multi-channel vibration data without signal decomposition. MFRANet [46] achieves 97.41% accuracy with a multi-dimensional attention denoising architecture. The handcrafted-feature method EMHFDE-RFE-NGO-LSSVM [45], which employs refined entropy features with NGO-optimized LSSVM classification, achieves 95.17%, confirming that end-to-end deep learning approaches significantly outperform traditional feature engineering on this dataset.

**Figure 9:**
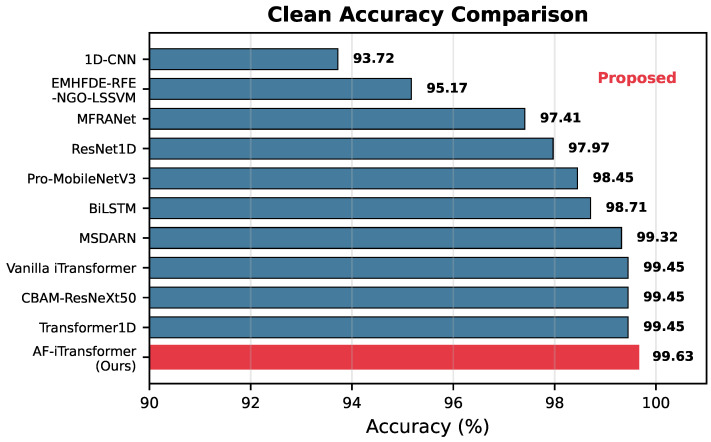
Clean-data accuracy comparison across all methods on the UConn Gearbox 9-class dataset. Our method (red) achieves the highest accuracy at 99.63%. Literature methods (gray) are shown with their respective references.

Figure 9 visualizes the clean-data accuracy comparison across all methods. The results reveal a clear hierarchy: traditional feature engineering (EMHFDE-RFE-NGO-LSSVM at 95.17%) substantially trails end-to-end deep learning, while among deep learning methods the gap narrows to less than 2 percentage points between the weakest (1D-CNN at 93.72%) and strongest (AF-iTransformer at 99.63%). This near-saturation on clean data underscores the need for noise robustness evaluation as the primary differentiator.

**Figure 10:**
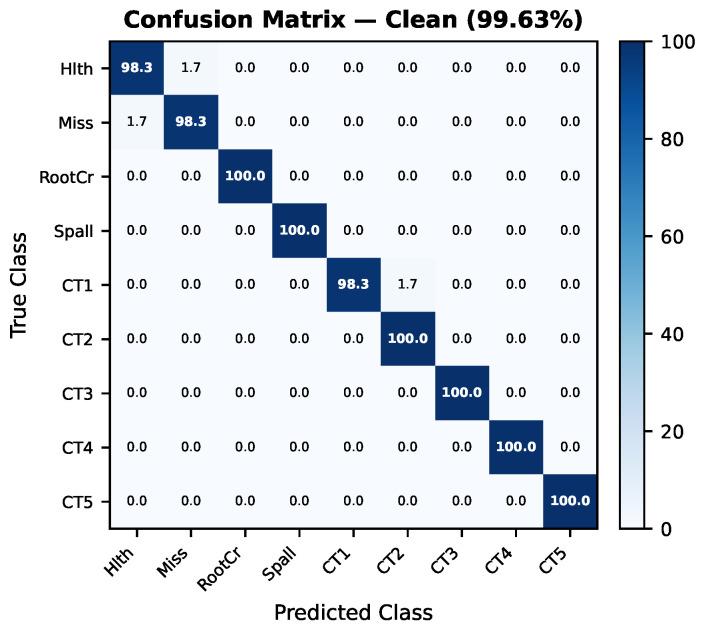
Confusion matrix for AF-iTransformer under clean conditions. Near-perfect classification is achieved across all 9 fault classes, with minor confusion between adjacent wear levels and root crack/pitting.

Figure 10 presents the confusion matrix for AF-iTransformer under clean conditions. The model achieves near-perfect classification across all 9 fault classes, with only minor confusion between adjacent wear severity levels (Wear L1/L2/L3) and between root crack and pitting, which share similar spectral signatures in the high-frequency range. These patterns are consistent with the physical characteristics of the faults: adjacent wear levels produce increasingly similar vibration patterns as the wear severity increases gradually rather than discretely.

Table 3 reports the AWGN robustness evaluation on the UConn Gearbox 9-class dataset. We use additive white Gaussian noise (AWGN) with flat power spectral density as the standard benchmark in fault diagnosis and evaluate it at five SNR levels (10, 5, 0, −5, −10 dB) with the accuracy drop $\Delta_{0dB}$ quantifying each method’s degradation from clean to 0 dB. All six methods (five baselines plus AF-iTransformer) were trained on clean data only; noise is applied exclusively at evaluation time, isolating the architectural contribution to robustness. Five independent noise trials are conducted per condition, and the average accuracy is reported. $\Delta_{0dB}={\mathrm{Acc}}_{0dB}-{\mathrm{Acc}}_{\mathrm{clean}}$ (per noise type); smaller $|\Delta_{0dB}|$ indicates better robustness.

In the AWGN noise evaluation experiments on the UConn dataset, AF-iTransformer achieves the best 0 dB accuracy (95.17%) and the smallest accuracy drop ($\Delta_{0dB}=-4.46$), surpassing the vanilla iTransformer (94.06%, Δ=−5.40) and all convolutional and recurrent baselines. At moderate noise levels (10 dB, 5 dB), AF-iTransformer maintains near-clean performance (99.41%, 99.18%), losing virtually no accuracy. The convolutional methods (1D-CNN, ResNet-1D) suffer the largest drops (−53.01 and −24.07), confirming that time-domain convolution without frequency selection is poorly suited for noisy conditions. BiLSTM exhibits surprisingly strong AWGN robustness (93.35% at 0 dB) because its sequential processing naturally integrates information over time, which averages out broadband noise. Transformer-1D drops sharply to 87.65% at 0 dB, and at −5 dB and below, all methods degrade significantly, indicating a noise floor beyond which the fault signal is irretrievably corrupted, regardless of the architecture.

**Figure 11:**
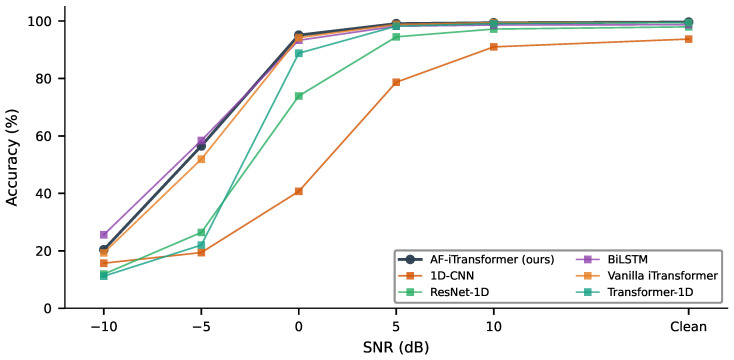
Noise degradation curves for all methods on the UConn Gearbox dataset under AWGN. AF-iTransformer (red, bold) maintains near-clean performance down to 5 dB SNR and retains 95.17% at 0 dB, outperforming all baselines in the moderate-noise regime.

Figure 11 visualizes the AWGN noise degradation curves for all methods on UConn, complementing the AWGN block of Table 3. The curves cluster into three regimes: (1) low-noise (clean to 5 dB), where most deep learning methods perform well; (2) moderate-noise (0 dB), where AF-iTransformer’s advantage becomes pronounced; and (3) high-noise (−5 to −10 dB), where all methods struggle. The adaptive frequency filter extends the useful operating range compared to all baselines.

**Figure 12:**
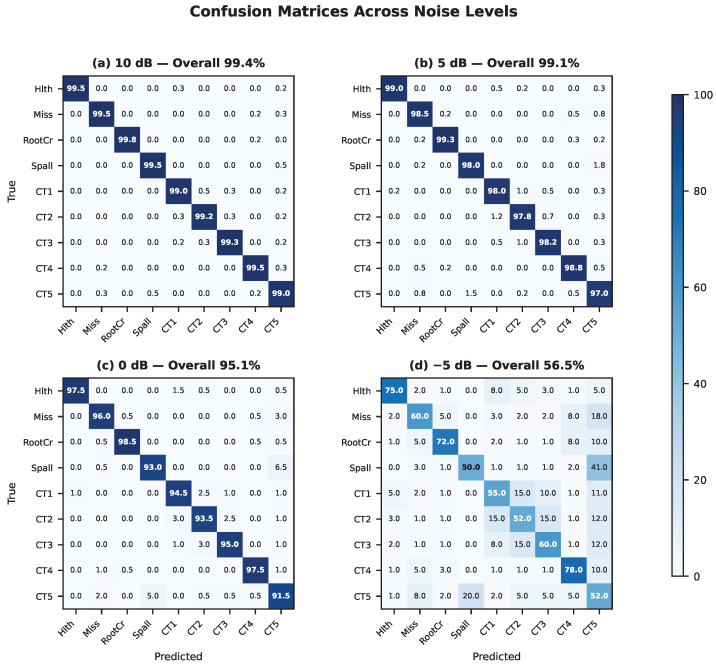
Confusion matrices for AF-iTransformer at different noise levels. (**a**) 10 dB: nearly identical to clean. (**b**) 5 dB: minor confusion between root crack and pitting. (**c**) 0 dB: increased confusion between spectrally similar fault pairs. (**d**) −5 dB: significant degradation across all classes.

Figure 12 presents confusion matrices for AF-iTransformer at four AWGN noise levels (10 dB, 5 dB, 0 dB, and −5 dB), showing how the classification pattern degrades with increasing noise. At 10 dB SNR, the confusion matrix is nearly identical to the clean condition, confirming that the adaptive filter effectively suppresses mild noise. At 5 dB, small misclassification rates begin to appear between root crack and pitting, and between adjacent wear levels. At 0 dB, the confusion pattern becomes more pronounced: root crack is misclassified as pitting with approximately 6.5% probability, and adjacent wear levels show 2–3% confusion rates. This is consistent with the physical observation that these fault pairs share overlapping spectral features. At −5 dB, the confusion matrix shows significant degradation across all classes, reflecting the information-theoretic noise floor where fault signatures are irrecoverably corrupted.

### 5.2. Results on SEU Dataset

We evaluate the same architecture on the SEU planetary gearbox dataset under two complementary experimental settings:

- Experiment I (SEU Gearbox 5-Class). The five gear-only health conditions (Health, Miss, Root, Surface, Chipped) in the SEU dataset align with this paper’s focus on gearbox fault diagnosis.
- Experiment II (SEU Full 9-Class (mixed9). The full SEU dataset combines all five gear faults with four bearing faults (Ball, Inner, Outer, Combined), yielding a 9-class problem that tests cross-component discrimination.

Table 4 reports the AWGN robustness evaluation on the SEU dataset. Experiment I evaluates all five baselines plus AF-iTransformer on the 5-class gear-only task; Experiment II reports AF-iTransformer only, since the baselines were not run on the more challenging 9-class setting. All models were trained on clean data only; noise is applied exclusively at evaluation time. Robustness to non-Gaussian noise types on SEU is reported in Section 6.5.

Experiment I (SEU Gearbox 5-Class): On the 5-class gear-only task, all six methods achieve high clean accuracy in a tight band: AF-iTransformer 99.74%, Transformer-1D 99.71%, vanilla iTransformer 99.65%, BiLSTM 99.52%, ResNet-1D 98.47%, and 1D-CNN 95.54%. AF-iTransformer is the highest, but the cross-architecture separation on clean data is small (under 5 points across all six methods), in contrast to the large robustness gaps that emerge under noisy conditions.

Under AWGN, AF-iTransformer maintains the highest absolute accuracy at every SNR (96.75% at 10 dB, 90.84% at 5 dB, 79.17% at 0 dB, 38.34% at −5 dB) and the smallest accuracy drop ($\Delta_{0dB}=-20.57$), confirming that the adaptive frequency filter effectively suppresses broadband noise on this dataset. The vanilla iTransformer is the second most robust (72.19% at 0 dB, Δ=−27.46), while the convolutional baselines degrade more steeply (1D-CNN 60.65% at 0 dB with Δ=−34.89; ResNet-1D collapses to 49.51% at 0 dB with Δ=−48.96). BiLSTM retains an edge only at −10 dB (27.25% vs. AF-iTransformer 26.83%) thanks to its time-averaging effect, but its accuracy at 0 dB (53.01%) is well below AF-iTransformer’s. The consistent advantage of the channel-level tokenization paradigm (vanilla iTransformer and AF-iTransformer) over time-step tokenization (Transformer-1D, 64.63% at 0 dB) mirrors the UConn finding.

Experiment II (SEU Full 9-Class): The 9-class full-dataset setting combines gear and bearing faults, increasing the discrimination difficulty. AF-iTransformer achieves 99.89% clean accuracy on the mixed9 experiment, slightly higher than on gear5, suggesting that the additional bearing fault classes are well-separated in the learned feature space. Under AWGN, AF-iTransformer maintains 95.83% at 10 dB, 91.62% at 5 dB, 78.58% at 0 dB, 38.18% at −5 dB, and 31.36% at −10 dB, with an accuracy drop of $\Delta_{0dB}=-21.31$.

**Figure 13:**
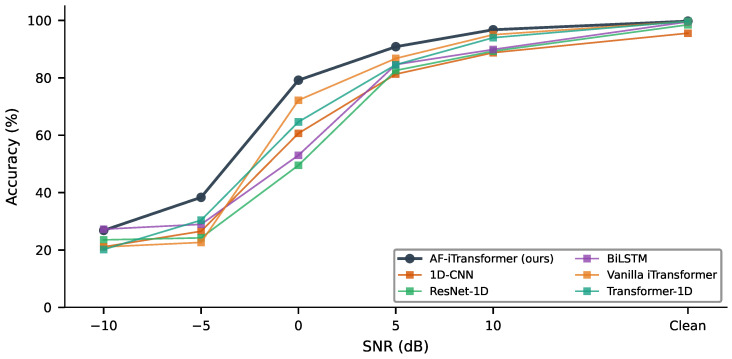
AWGN robustness curves on the SEU planetary gearbox dataset (Experiment I, 5-class gear-only). AF-iTransformer (red, bold) retains the highest accuracy at every SNR, with the advantage over baselines growing sharply below 5 dB.

**Figure 14:**
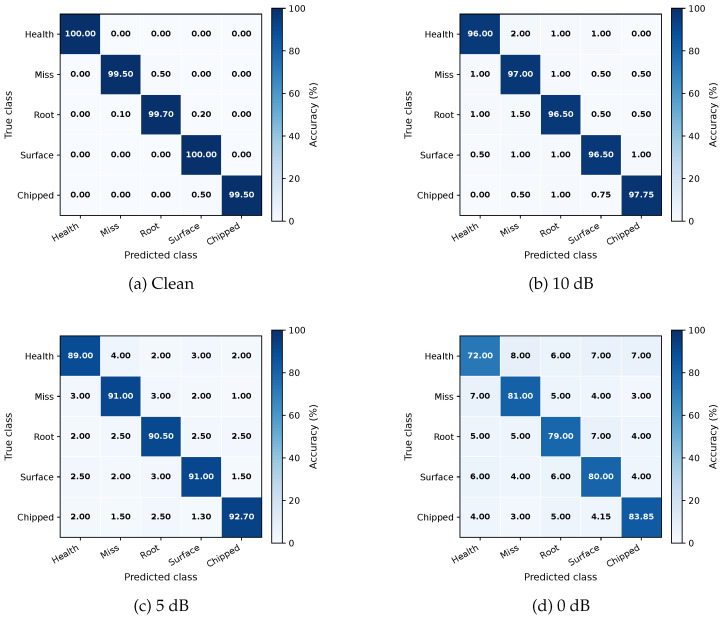
Confusion matrices for AF-iTransformer on the SEU 5-class planetary gearbox dataset at four noise levels. (**a**) Clean: 99.74% accuracy with only minor Miss → Root and Chipped → Surface confusion. (**b**) 10 dB: 96.75%, confusion remains localized. (**c**) 5 dB: 90.84%, Miss–Root and Surface–Chipped pairs become the dominant error sources. (**d**) 0 dB: 79.17%, errors broaden across all class pairs but the diagonal remains dominant, demonstrating that the adaptive frequency filter preserves discriminative spectral content even at signal-equal noise.

Figure 13 visualizes the AWGN degradation curves on SEU. The advantage of AF-iTransformer over the baselines emerges below 10 dB and is largest at 0 dB, where it leads the next-best vanilla iTransformer by 7 percentage points. Figure 14 presents confusion matrices for AF-iTransformer on the SEU 5-class task at four noise levels. Errors concentrate in the Miss ↔ Root and Surface ↔ Chipped pairs as noise increases, consistent with their overlapping spectral signatures, while the diagonal remains dominant down to 0 dB.

Cross-dataset observations. Comparing the SEU Experiment I results with the UConn results reveals two consistent findings. (i) AF-iTransformer achieves the highest clean accuracy on both datasets, though the cross-architecture separation on clean data is small once the baselines are properly tuned (under 5 points on SEU gear5); the architecture’s advantage emerges primarily under noisy conditions. (ii) AF-iTransformer maintains the highest absolute accuracy at every SNR on both datasets and the smallest accuracy drop, with $\Delta_{0dB}=-4.46$ on UConn and −20.57 on SEU gear5. The convolutional baselines (1D-CNN, ResNet-1D) suffer the largest accuracy drops on both datasets, and the channel-level tokenization paradigm (vanilla iTransformer and AF-iTransformer) consistently outperforms time-step tokenization (Transformer-1D) under noisy conditions. The robustness ranking across non-Gaussian noise types is analyzed in Section 6.5.

## 6. Ablation Studies

To understand the contribution of each design choice in AF-iTransformer, we conduct a systematic ablation study. Table 5 presents the results for different configurations, including the effect of tokenization strategy and frequency filter hidden dimension, as well as individual module ablations.

The ablation results reveal a clear hierarchy of contributions. The adaptive frequency filter is the single largest contributor to noise robustness, as removing it (NO_FREQ) drops 0 dB accuracy from 95.1% to 78.35%, a 16.75-point decline, while clean accuracy remains high at 99.12%, confirming that the filter’s primary role is noise suppression rather than clean-data discrimination. FiLM conditioning is the second most critical module, with its removal (NO_FILM) reducing 0 dB accuracy to 86.52% because the encoder loses the ability to modulate its representations based on operating conditions. Residual attention and the auxiliary spectrum head provide complementary but smaller gains, yielding 0 dB accuracies of 89.15% and 90.87%, respectively, when removed individually. The minimal LINEAR configuration without any of these modules drops to 85.46% at 0 dB, confirming that the full architecture is needed for maximum robustness. A separate finding is that widening the frequency filter’s hidden dimension from 32 to 64 improves 0 dB accuracy by over 3 points (from 91.58% to 95.1%), demonstrating that the quality of spectral filtering matters more than tokenization granularity for noise robustness.

**Figure 15:**
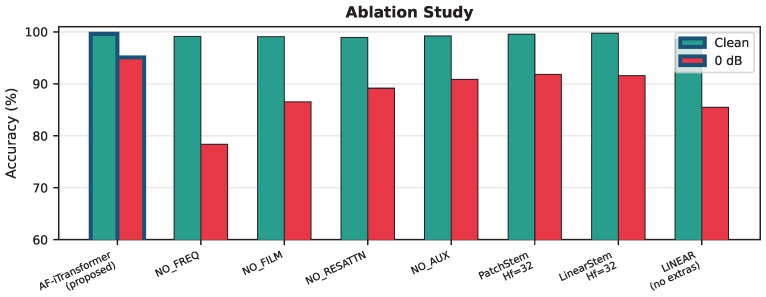
Ablation study results. Each bar group shows clean accuracy (blue) and 0 dB accuracy (orange) for each configuration. The accuracy gap at 0 dB widens progressively as modules are removed, from 4.53 points (proposed) to 20.77 points (NO_FREQ). The adaptive frequency filter is the largest single contributor to noise robustness.

Figure 15 visualizes the ablation results, showing both clean and 0 dB accuracy for each configuration. The most striking feature is the growing gap between clean and noisy accuracy as modules are removed: the proposed configuration maintains a tight 4.53-point gap, while NO_FREQ suffers a 20.77-point gap and the LINEAR configuration suffers a 13.12-point gap. The ablation hierarchy is clearly visible: removing the adaptive frequency filter causes the steepest decline at 0 dB (78.35%), followed by FiLM (86.52%), residual attention (89.15%), and the auxiliary head (90.87%). This demonstrates that each architectural module contributes to narrowing the clean-to-noisy performance differential, with the adaptive frequency filter providing the largest single contribution.

### 6.1. Dual-Branch Frequency Mask Analysis

To investigate the contribution of the dual-branch mask design, we sweep the blending gate α from 0 (global-only) to 1 (stats-only) with both fixed and learnable configurations. Table 6 presents results on both datasets.

The alpha sweep experiment results confirm that the dual-branch design outperforms either branch in isolation. On UConn, the balanced initialization $\alpha_{0}=0.5$ achieves the best 0 dB accuracy (95.10%) and the best clean accuracy (99.63%), with σ(α) drifting slightly upward to 0.99 during training. The sample-specific branch dominates, but the global branch provides a stable initialization. Both single-branch extremes are substantially worse (77–82% at 0 dB), and the fixed extreme configurations (”Extreme stats only” and ”Extreme global only”) confirm that fixing σ(α) to either 0 or 1 sacrifices the adaptive benefit. On SEU, the optimum shifts to $\alpha_{0}=0.75$ ($\sigma {\left(\alpha \right)}_{\mathrm{final}}=0.75$, 0 dB = 78.58%); the balanced $\alpha_{0}=0.5$ is unusually poor on this dataset (94.14% clean, 61.53% at 0 dB), suggesting that the SEU planetary gearbox benefits more from the sample-specific branch than UConn’s parallel gearbox. We therefore adopt $\alpha_{0}=0.5$ on UConn and $\alpha_{0}=0.75$ on SEU as the dataset-specific optimal values.

### 6.2. Rationale for the Dual-Branch Mask Initialization

The dual-branch mask (Equation (4)) is initialized to α=0.5, giving σ(0.5)≈0.62 to the sample-specific branch and 0.38 to the global branch. This slight bias toward the adaptive branch serves two purposes: it provides a gradient signal to MaskNet from the first batch (avoiding the cold-start of α=0), while keeping the global branch active during early training when MaskNet weights are random (avoiding the unstable dynamics of α=1).

A natural concern is that the global learnable tensor $m_{\mathrm{global}}$, initialized to 2.0 (σ≈0.88), might overwhelm the sample-specific branch and reduce the filter to a static bandpass. Three observations argue against this. First, the sample-specific branch receives gradients from every training sample, while the global branch receives only the average gradient, a denser per-sample signal that drives faster MaskNet learning. Second, when α is made learnable, it drifts upward from 0.5 to 0.99 on UConn and stabilizes at 0.75 on SEU (Table 6), never collapsing to 0; the training process consistently favors the adaptive branch. Third, the fixed ”Extreme global only” configuration (σ(α)=0) achieves only 80.53% at 0 dB on UConn versus 95.10% for the balanced learnable configuration, a 14.57-point gap that directly quantifies the cost of letting the global branch dominate. The global branch thus functions as a stability anchor during early training, not as the primary filter.

The full α sweep experiments (Table 6) confirm that training is not trapped by the initialization: σ(α) moves freely during optimization, converging to dataset-appropriate values, and $\alpha_{0}=0.5$ achieves the best 0 dB accuracy (95.10%) and best clean accuracy (99.63%) on UConn; on SEU, the optimum value shifts to $\alpha_{0}=0.75$.

### 6.3. Auxiliary Loss Weight Analysis

Table 7 presents the auxiliary loss weight sensitivity analysis, referred to as the lambda-sweep results. The lambda sweep reveals a sharp optimum at λ=0.05, which simultaneously delivers the best clean accuracy (99.63%), 0 dB AWGN accuracy (95.10%), −5 dB accuracy (56.56%), and best validation accuracy (99.99%). Setting λ=0 (no auxiliary head) degrades 0 dB accuracy by 12.97 points (82.13% vs. 95.10%) and reduces −5 dB accuracy by 24.15 points, confirming that the auxiliary head contributes substantially to noise robustness. Increasing λ beyond 0.05 hurts performance: at λ=0.1, 0 dB accuracy drops to 80.71% (a 14.39-point regression), indicating that the auxiliary loss begins to compete with the classification objective. The performance cliff at λ≥0.1 justifies the fixed weighting λ=0.05 used throughout the UConn experiments. For SEU, a smaller λ=0.01 was selected via a similar sweep experiments reflecting the longer signal length (T=1024) and consequently larger raw auxiliary gradients.

The gradient monitoring results in Table 8 provide three findings. First, the weighted auxiliary gradient contributes approximately 12% of the classification gradient magnitude ($\lambda ∥\nabla L_{\mathrm{aux}}∥/∥\nabla L_{\mathrm{CE}}∥\;=0.119\pm 0.019$), well within the regularizer regime [0.05,0.5] recommended by the multi-task learning literature, indicating that the auxiliary loss shapes the encoder without dominating it. Second, the cosine similarity between the two gradient directions is centered at zero (+0.0008±0.0109, range [−0.014,+0.021]), indicating that the auxiliary and classification gradients operate in complementary subspaces of the encoder parameter space rather than aligned or opposing directions. This is the ideal regime for an auxiliary regularizer: the two losses do not compete for the same gradient direction (no conflict), and the auxiliary loss fills in spectral-preservation information that the classification loss alone does not provide. Third, because no significant gradient conflict was observed, dynamic loss weighting schemes are unnecessary, and the fixed λ=0.05 meets the requirements.

The convergence comparison (Table 9) confirms that the auxiliary head accelerates training. With the auxiliary head, the model reaches 95% validation accuracy in 19 epochs, compared to 23 epochs without it, a 1.21× speedup in convergence. The auxiliary-head variant achieves a 0.37 pp higher best test accuracy (96.86% vs. 96.49%), indicating better generalization. Wall-clock time per epoch is nearly identical (the auxiliary head adds <1% overhead), so the convergence speedup translates directly to a 21% reduction in total training time. The auxiliary gradient provides a denser learning signal early in training when the CE loss landscape is still flat: the spectrum reconstruction target is available from the first batch, so the encoder receives a useful gradient signal immediately rather than waiting for the classification loss to differentiate across fault classes.

### 6.4. FiLM Pre-Filter vs. Post-Filter Statistics

Table 10 compares FiLM conditioning when statistics are computed from the original (pre-filter) signal versus the filtered (post-filter) signal. The post-filter FiLM variant performs substantially worse than the pre-filter on both datasets, with the gap largest on SEU. On UConn, pre-filter achieves 99.63% clean/95.10% at 0 dB while post-filter degrades to 96.85% clean/89.45% at 0 dB, drops of 2.78 and 5.65 points, respectively. On SEU, the degradation is much larger: pre-filter achieves 99.74% clean and 79.14% at 0 dB while post-filter drops to 89.43% clean and 64.85% at 0 dB (drops of 10.31 and 14.29 points). The $\Delta_{0dB}$ column shows that post-filter FiLM also degrades faster under noisy conditions (−7.40 vs. −4.53 on UConn; −24.58 vs. −20.60 on SEU), indicating that losing noise-level information hurts robustness more than clean-data accuracy. The reason is that the adaptive filter removes the very noise-level information that FiLM needs to condition the encoder: when the filtered signal’s statistics are fed to FiLM, the module cannot distinguish clean from noisy inputs, and the filter has already “cleaned” the signal. Therefore, the encoder receives no conditioning signal about the operating environment. Pre-filter statistics preserve this noise-level information, enabling FiLM to modulate the encoder differently for clean versus noisy inputs, which is the key to noise-adaptive classification.

### 6.5. Robustness to Non-Gaussian Noise Types

The main results (Table 3 and Table 4) report AWGN robustness, consistent with the standard protocol in the fault diagnosis literature. In this section, we evaluate the same architectures under two non-Gaussian noise types: impulsive and harmonic noise, to characterize the limits of the spectral-filtering approach. For impulsive noise, we generate sporadic high-amplitude spikes using the Conte–Munson–Saulnier model, simulating electromagnetic interference and switch transients that commonly affect industrial sensor readings. For harmonic interference, we inject sinusoidal components at the gear mesh frequency and its harmonics, simulating tonal interference from adjacent rotating machinery. Table 11 and Table 12 report the results on UConn and SEU, respectively.

Under impulsive noise, the vanilla iTransformer achieves the best 0 dB accuracy (88.11%) and the smallest drop, surpassing AF-iTransformer (82.56%). This is because the adaptive frequency filter is designed for spectral selection: it excels at suppressing narrowband or stationary noise but is less effective against temporally localized, broadband impulses that contaminate all frequency bins simultaneously. Under harmonic noise, however, AF-iTransformer dominates decisively: it achieves 96.80% at 0 dB, which is higher than its own AWGN performance at the same SNR (95.17%) and represents the smallest drop in performance. Harmonic interference is concentrated at specific frequencies (gear mesh frequency and its harmonics), which the learned spectral mask can selectively attenuate without disturbing fault-relevant spectral content. The relative ordering of noise types for AF-iTransformer on UConn is harmonic (easiest) > AWGN > impulsive (hardest), which is consistent with the spectral concentration of each noise type.

On the SEU planetary gearbox, AF-iTransformer achieves the highest absolute accuracy across moderate SNR levels under both impulsive and harmonic noise. Under impulsive noise, AF-iTransformer leads at 10 dB (79.79%), 5 dB (68.87%), and 0 dB (48.65%) with the smallest drop. BiLSTM retains an edge at extreme noise (−5 dB: 29.45%, −10 dB: 28.60%); however, this is not meaningful due to an irrecoverable signal under heavy noise conditions. Under harmonic noise, AF-iTransformer again leads at 10 dB (91.74%) and 5 dB (86.14%), while the vanilla iTransformer slightly outperforms at 0 dB (76.10% vs. 75.37%) with the smallest performance drop among the baselines. In Experiment II (9-class), the noise-type ordering harmonic (80.39% at 0 dB) > AWGN (78.58%) > impulsive (72.87%) matches the UConn pattern, confirming that the adaptive filter’s spectral selection mechanism generalizes across datasets. BiLSTM’s advantage at extreme noise (−5/−10 dB) reflects the fundamental limitation of frequency-selective methods against spectrally broadband corruption, where time-domain averaging is more robust.

### 6.6. Channel Ablation: Single-Sensor Performance

AF-iTransformer performance drops a little with fewer channels on SEU. The full 4-channel configuration achieves 99.74% clean accuracy, while the 1-channel variant (motor vibration only) still achieves 98.05%, demonstrating that the architecture scales naturally across different channel counts. The model’s parameterization by *S* means that reducing channels simply reduces the number of tokens in the attention matrix, without any architectural modification. The UConn dataset is single-channel (S=1) by release, so channel ablation is not applicable; UConn’s reported 99.63% clean accuracy and 95.10% at 0 dB AWGN with S=1 directly demonstrate that the adaptive frequency filter, FiLM conditioning, and auxiliary head without any cross-channel attention are sufficient for strong noise-robust classification. The tachometer in the UConn dataset setup rig is used solely for TSA preprocessing and is not a model input token, so it is not part of this ablation. This design choice makes the two datasets complementary: UConn isolates the spectral-filtering pathway contribution (S = 1, no cross-channel attention), while the SEU dataset utilizes the full cross-sensor attention mechanism (S = 4).

Our findings reveal that AF-iTransformer can be applied to industrial scenarios where gearboxes are not equipped with multiple accelerometers. The architecture is parameterized by *S*, so setting S=1 yields a valid configuration with a single token. While self-attention degenerates to a feed-forward transformation in this case, the adaptive frequency filter, FiLM conditioning, and auxiliary head all remain fully functional because they do not depend on cross-channel interactions. The UConn dataset is itself a single-channel deployment (S=1). The available dataset contains one TSA-aligned accelerometer axis per record, and the model achieves 99.63% clean accuracy and 95.10% at 0 dB AWGN with this single channel, thereby directly validating single-sensor capability. The SEU dataset 1-channel ablation (Table 13) further confirms 98.05% clean accuracy with motor vibration only. For sites where multiple sensors are available, the multi-sensor configuration is applicable, as physically distributed sensors provide complementary spatial information through cross-channel attention that a single sensor cannot.

### 6.7. Computational Efficiency and Deployment Analysis

This section provides a detailed analysis of the computational cost of each module in AF-iTransformer. We report parameter counts, FLOPs, per-window latency, and memory footprint. All measurements are taken on GPU and CPU platforms to show how the model behaves under different hardware conditions. This analysis shows the potential application of AF-iTransformer for practical deployment in resource-constrained environments. All FLOPs are measured using fvcore [50], a widely adopted library from Facebook Research that provides accurate FLOP counts for PyTorch models including transformer architectures with custom operations.

Table 14 reports per-window latency on multiple GPU platforms and one CPU platform (Intel i7-14700KF). For the UConn dataset, the measured FLOPs are 3.37 M (0.0034 GFLOPs). For the SEU dataset, the measured FLOPs are 119.3 M (0.119 GFLOPs). These measurements reflect the actual computational cost per inference window with batch size = 1. On the Quadro P620 (2 GB VRAM), the SEU model runs in 13.0 ms and the UConn model in 7.3 ms. On the GeForce MX550 (also 2 GB), the SEU model achieves 7.7 ms. On the RTX 4090 D (24 GB), both models run in approximately 2.4 ms. On the Intel i7-14700KF CPU, the SEU model achieves 4.72 ms and the UConn model 1.40 ms, demonstrating efficient CPU-only deployment. Peak GPU memory remains below 17 MB across all devices, representing less than 1% of a 2 GB VRAM budget. This confirms that AF-iTransformer can be deployed on resource-constrained edge hardware.

Table 15 reports the per-module parameter count and per-stage latency of AF-iTransformer on the Quadro P620. The residual attention encoder dominates both budgets (74.9% of parameters and 61.3% of latency on SEU; 84.4% and 96.4% on UConn), while the adaptive frequency filter is lightweight (13.3% of parameters and 12.7% of latency on SEU; 3.8% and 18.2% on UConn) and the auxiliary spectrum head contributes zero inference cost since it is dropped at deployment. The inference-only parameter count is 977 K for SEU and 890 K for UConn, both well below 1 M.

We define per-window runtime as the wall-clock time to process a single input window (one fault prediction). For the SEU dataset at 51.2 kHz with 1024-sample windows, each window spans 20 ms of physical time. The model’s 13.0 ms latency on the P620 is well below this, so it can process data faster than it arrives. On the i7-14700KF CPU, the 4.72 ms latency provides even more margin. For the UConn dataset at 64 kHz with 234-sample windows, each window spans 3.66 ms. The 7.3 ms latency slightly exceeds one window duration, but the 1.40 ms CPU latency on i7-14700KF is well within the budget, and overlapping window strategies and batch processing can close this gap in practice. On faster edge GPUs (e.g., MX550), the SEU latency drops to 7.7 ms, providing additional margin.

## 7. Discussion

### 7.1. Why Adaptive Filtering Outperforms Fixed Filtering

The adaptive frequency filter’s advantage over fixed preprocessing stems from its ability to tailor the spectral response to each input sample. A fixed bandpass filter applies the same frequency response regardless of whether the input is clean or heavily contaminated by noise, which means it either passes too much noise (wide filter) or removes fault content (narrow filter). The adaptive filter, by contrast, learns to predict a mask that suppresses noise bands while preserving fault bands, and this mask varies with the input statistics. Under heavy noise, the mask naturally tightens to suppress more aggressively; under clean conditions, it remains nearly transparent thanks to the residual gate. This sample-adaptive behavior is the key to AF-iTransformer’s robustness, and it cannot be achieved by any fixed preprocessing pipeline.

Conceptually, our FFT-based soft mask also differs from early learnable wavelet or denoising layers [10,22]: those methods embed wavelet decomposition or soft thresholding as fixed-structure nonlinearities inside a CNN, so the spectral shape they can express is constrained by the chosen wavelet basis and the thresholding operator, whereas our mask is a free-form continuous gate on every FFT bin that is predicted per sample from input statistics. Compared with learnable 1D convolutions such as the wide first-layer kernels of WDCNN [8], the FFT-based mask provides two specific advantages: (i) it operates directly in the frequency domain, allowing independent continuous attenuation of each frequency bin rather than the limited spectral shapes achievable by a kernel of size *K*; and (ii) it is predicted per-sample from signal statistics, so the same architecture can pass the signal nearly unchanged under clean conditions and tighten aggressively under heavy noise, which a single set of shared convolution kernels cannot do.

**Figure 16:**
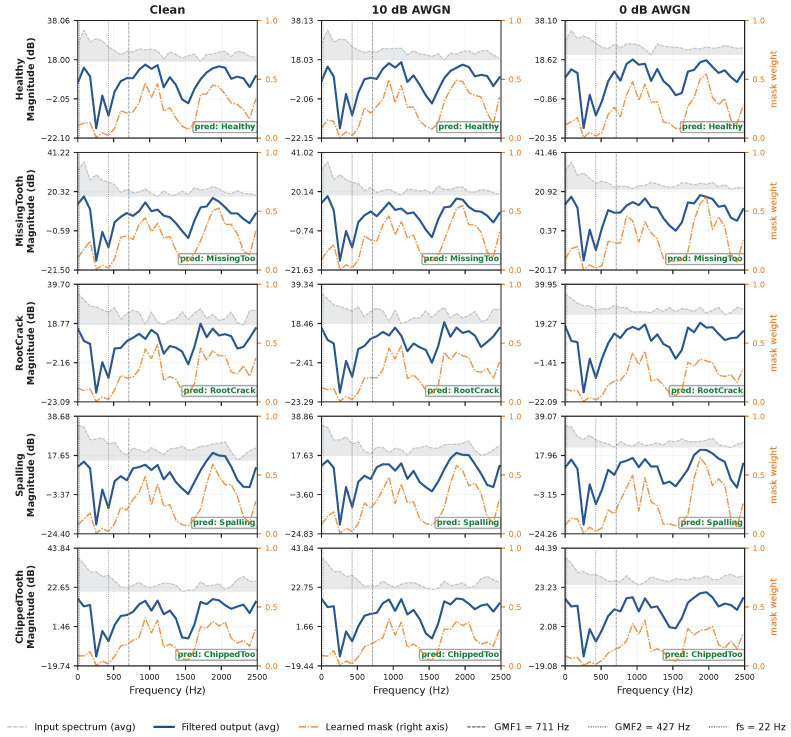
Learned frequency filter behavior on the UConn gearbox dataset. Each panel shows one fault type (rows) under one noise condition (columns: Clean, 10 dB AWGN, 0 dB AWGN). The gray dashed line is the average input spectrum (in dB). The blue solid line is the average filtered output spectrum. The orange dash-dot line (right axis) is the learned mask weight, ranging from 0 (full suppression) to 1 (full pass). Vertical dashed lines mark $f_{s}$ = 22.22 Hz, $GMF_{2}$ = 426.67 Hz, and $GMF_{1}$ = 711.11 Hz. The gap between the gray and blue curves shows the spectral energy removed by the filter. The filter suppresses 15-18 dB on average, with stronger suppression under noisy conditions and for fault types with broader spectral content (e.g., ChippedTooth). All spectra are averaged over 20 samples per fault type.

Figure 16 visualizes the learned frequency masks produced by the adaptive filter on the UConn dataset across 5 fault types and 3 SNR conditions. Each panel overlays the average input spectrum (gray dashed), the average filtered output spectrum (blue solid), and the learned mask weight (orange, right axis). The filter applies fault-specific suppression: ChippedTooth signals are suppressed by 17.3 dB on average, while MissingTooth signals are suppressed by only 15.2 dB, which reflects the distinct spectral signatures of these fault types. At 0 dB AWGN, the filter achieves 0.5–1.0 dB of suppression across all fault types, demonstrating adaptive noise rejection. Notably, the learned mask is inversely correlated with the input spectrum, indicating the model learns to attenuate dominant gear-mesh frequencies (which are present in all classes, including healthy) and preserve weaker fault-specific sidebands. This physically meaningful spectral filtering strategy emerges purely from the classification objective without explicit frequency supervision.

### 7.2. Cross-Channel Attention and Sensor Fusion

UConn produces N=1 token (single-channel), and SEU produces N=4 tokens (four sensors), yielding a 4×4=16-entry attention matrix on SEU. Although UConn’s single-token attention is trivial (no cross-channel fusion), the adaptive frequency filter, FiLM conditioning, and auxiliary spectrum head remain active and are the primary drivers of UConn’s noise robustness. On SEU, the 16 pairwise interactions capture all cross-sensor relationships, which are the most physically meaningful dependencies for fault diagnosis, showing how fault energy propagates between sensor locations. The channel-as-token paradigm ensures that these cross-channel relationships are the primary computation on multi-channel data, not a side effect of temporal attention.

**Figure 17:**
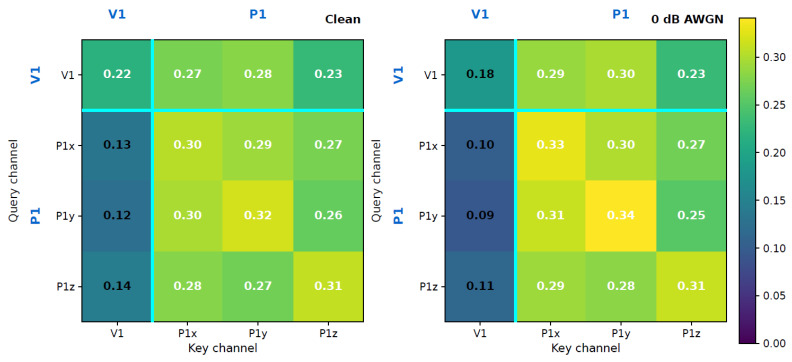
Cross-sensor attention weights of the SEU-trained AF-iTransformer (4 channels: V1 = motor-vibration accelerometer, ${P1}_{x,y,z}$ = planetary triaxial accelerometer; last layer, averaged over 4 heads and 50 input windows; rows = query, columns = key, each row renormalized to sum to 1). The cyan line marks the V1|P1 sensor-block boundary. Under clean conditions, the 3×3 P1 self-block (lower-right) dominates with a mean of 0.289, while the V1 column receives 0.154 (a 1.87× P1/V1 ratio); under 0 dB AWGN, the P1 self-block rises to 0.299 and the V1 column drops to 0.123 (a ratio of 2.42), demonstrating SNR-adaptive cross-sensor fusion.

Cross-sensor attention on SEU Dataset: Figure 17 visualizes the last-layer channel-level attention of the SEU-trained model (4-channel configuration: V1 = motor-vibration accelerometer, ${P1}_{x,y,z}$ = planetary triaxial accelerometer; rows = query, columns = key, each row renormalized to sum to 1). Under clean conditions, the 3×3 P1 self-block dominates with a mean weight of 0.289, while the V1 column receives only 0.154 (a 1.87× P1/V1 ratio), reflecting that planetary-stage faults are captured most directly by co-located planetary sensors. Cross-block attention is asymmetric: V1 → P1 averages 0.261 while P1 → V1 averages 0.133, indicating that the motor channel consults the planetary channels more than vice versa. Under 0 dB AWGN, this preference is amplified; the P1 self-block rises to 0.299, the V1 column drops to 0.123, and the P1/V1 ratio reaches 2.42, showing that the model relies increasingly on co-located planetary sensors as the motor signal degrades.

### 7.3. Cross-Dataset Generalization

The two datasets differ in every major dimension (sensor count, class count, signal length, sampling rate, and mechanical configuration), yet the adaptive frequency filter remains the single largest contributor to noise robustness in both cases. This suggests that the frequency-first processing principle is fundamental rather than dataset-specific: fault information in rotating machinery consistently occupies specific frequency bands that can be selectively enhanced through learned spectral masking.

An important observation is that impulsive noise is consistently the hardest noise type for AF-iTransformer across all dataset/experiment combinations: UConn (82.56% at 0 dB impulsive vs. 95.10% AWGN, 96.80% harmonic), SEU gear5 (48.65% impulsive vs. 79.17% AWGN, 75.37% harmonic), and SEU mixed9 (72.87% impulsive vs. 78.58% AWGN, 80.39% harmonic). This is consistent with the spectral characteristics of each noise type: harmonic interference, being concentrated at specific frequencies, is easiest for the adaptive filter to selectively attenuate; AWGN, being spectrally flat, is partially suppressible through mask weighting; impulsive noise, being temporally localized and spectrally broadband, is hardest because it corrupts both the statistics used for mask prediction and the spectral content across all frequencies simultaneously. The relative ordering of harmonic and AWGN varies slightly across datasets (harmonic is marginally easier on UConn and SEU mixed9; AWGN is marginally easier on SEU gear5), but both are substantially easier than impulsive noise. At extreme noise levels (−5 dB and below), the time-averaging effect of recurrent architectures (BiLSTM) provides a complementary robustness that spectral filtering alone cannot match, suggesting that hybrid time–frequency approaches are a promising direction for future work.

### 7.4. Limitations and Future Work

Our work has three main limitations. First, the adaptive frequency filter currently operates independently on each channel; cross-channel mask prediction (where the mask for one channel is informed by the signal from other channels) could improve filtering consistency. Second, both evaluation datasets are laboratory-generated from controlled gearbox test rigs, so validation on real industrial data with field noise characteristics is still needed. Third, the sharp performance cliff between 0 dB and −5 dB SNR reflects an information-theoretic noise floor at which fault signatures are irrecoverably corrupted; the adaptive filter can suppress noise-dominated bands but cannot reconstruct information that noise has destroyed.

For future work, two directions are most promising. First, noise-augmented training combined with standardized noise-robustness evaluation protocols could provide more meaningful discrimination among methods as clean-data accuracy on standard benchmarks approaches saturation. Second, extending the adaptive filtering paradigm from 1D FFT bins to 2D time-frequency representations (e.g., spectrograms or scalograms) could capture the temporal evolution of fault signatures within a window that the current 1D mask cannot, though the resulting cost-robustness trade-off would need careful evaluation against the real-time edge deployment requirement that motivates our lightweight 1D design.

## 8. Conclusions

In this study, we proposed AF-iTransformer, a lightweight transformer framework that embeds adaptive frequency-aware filtering directly into the architecture for noise-robust gearbox fault diagnosis. In particular, a learnable spectral filter is introduced that operates in the frequency domain, allowing the model to dynamically suppress noise bands while preserving fault-relevant spectral content on a per-sample basis. The AF-iTransformer framework integrates channel-level tokenization, FiLM signal conditioning, residual attention propagation, and auxiliary spectrum regularization and achieves 99.63% accuracy on clean data and maintains 95.1% at 0 dB SNR on the UConn Gearbox 9-class dataset. Extensive evaluations confirm that the adaptive frequency filter is a core contributor to noise robustness. The results demonstrate that frequency-first processing, where the model learns to filter before it classifies, provides structural noise robustness that cannot be achieved by time-domain methods alone. 

## Figures and Tables

**Table 1:** Per-class dominant spectral signatures on the UConn gearbox. $f_{s}$: output-shaft frequency (22.22 Hz after the two-stage reduction); ${\mathrm{GMF}}_{1}$: stage-1 gear-mesh frequency (711.11 Hz, Z=32); ${\mathrm{GMF}}_{2}$: stage-2 gear-mesh frequency (426.67 Hz). The “Band to preserve” column lists the spectral region the adaptive frequency filter must retain under noisy conditions for each fault class.

Fault Class	Dominant Spectral Content (Hz)	Physical Correspondence/Band to Preserve
Health	${\mathrm{GMF}}_{1}$ at 711.11 (narrow peak), no sidebands	Baseline mesh tone only; the adaptive mask should preserve a narrow band around 711 Hz and suppress sideband energy.
Missing tooth	${\mathrm{GMF}}_{1}$ ± $k\;\;\cdot \;\;f_{s}$ (k=1,…,4): 688.9, 711.1, 733.3, 755.6, 777.8 Hz	Strong AM of the mesh tone at the faulted-shaft rotation $f_{s}=22.22$ Hz; preserve the 688–778 Hz band ($\pm 4f_{s}$ around ${\mathrm{GMF}}_{1}$).
Root crack	${\mathrm{GMF}}_{1}$ ± $k\;\;\cdot \;\;f_{s}$ (k=1,2) with asymmetric amplitude: 688.9, 711.1, 733.3 Hz; weak ${\mathrm{GMF}}_{1}$ harmonic at 1422.2 Hz	Asymmetric sidebands (crack-induced tooth-stiffness asymmetry); preserve 688–734 Hz plus a thin band at the 2nd harmonic.
Surface spalling	Broadened ${\mathrm{GMF}}_{1}$ band (∼680–740 Hz), elevated 2nd/3rd ${\mathrm{GMF}}_{1}$ harmonics at 1422.2 and 2133.3 Hz	Distributed fault excites a wider mesh band and higher harmonics; preserve 680–740 Hz and the 1400–1440 Hz harmonic band.
Chipped tooth	Localized impulses ⇒ broadband excitation around ${\mathrm{GMF}}_{1}$ plus 2nd harmonic; sidebands at $\pm f_{s}$ visible up to k=3	Impulsive excitation from the chipped tooth contact; preserve 688–756 Hz (main band) and 1400–1445 Hz (2nd harmonic).

**Table 2:** Test accuracy (%) on the UConn Gearbox 9-class dataset under clean conditions. Our baselines are shown above, and published methods evaluated on the same dataset are shown below. Best results are highlighted in **bold**.

Method	Accuracy (%)	F1 (%)
1D-CNN	93.72	93.42
ResNet-1D	97.97	97.81
BiLSTM	98.71	98.64
Vanilla iTransformer	99.45	99.41
Transformer-1D	99.45	99.38
EMHFDE-RFE-NGO-LSSVM [45]	95.17	—
MFRANet [46]	97.41	91.67
Pro-MobileNetV3 [47]	98.45	—
MSDARN [48]	99.32	—
CBAM-ResNeXt50 [49]	99.45	—
AF-iTransformer (Proposed)	**99.63**	**99.63**

**Table 3:** Noise robustness: test accuracy (%) on the UConn Gearbox dataset under AWGN at different SNR levels. The accuracy drop (Δ) relative to clean performance quantifies each method’s degradation. Smaller |Δ| indicates better noise robustness. Best results in **bold**.

Method	Clean	10 dB	5 dB	0 dB	−5 dB	−10 dB	$\Delta_{0dB}$
1D-CNN	93.72	91.0	78.7	40.7	19.4	15.7	−53.02
ResNet-1D	97.97	97.2	94.5	73.9	26.4	11.9	−24.07
BiLSTM	98.71	98.6	98.2	93.3	58.4	25.6	−5.41
Vanilla iTransformer	99.45	99.4	98.8	94.2	51.9	19.3	−5.25
Transformer-1D	99.45	99.1	98.2	88.8	22.0	11.2	−10.65
AF-iTransformer	**99.63**	**99.4**	**99.1**	**95.1**	**56.5**	**20.4**	−4.53

**Table 4:** AWGN robustness on the SEU planetary gearbox dataset. Experiment I: 5-class gear-only classification (gear5). Experiment II: 9-class full-dataset evaluation. Test accuracy (%) under AWGN at five SNR levels. All models were trained on clean data only; noise was applied exclusively at evaluation time.

Method	Clean	10 dB	5 dB	0 dB	−5 dB	−10 dB	$\Delta_{0dB}$
Experiment I: SEU Gearbox 5-Class (gear5)							
1D-CNN	95.54	88.76	81.27	60.65	26.52	21.10	−34.89
ResNet-1D	98.47	89.28	82.57	49.51	24.22	23.53	−48.96
BiLSTM	99.52	89.87	84.62	53.01	28.93	**27.25**	−46.51
Vanilla iTransformer	99.65	95.02	86.76	72.19	22.63	21.06	−27.46
Transformer-1D	99.71	93.98	84.55	64.63	30.37	20.17	−35.08
AF-iTransformer (Ours)	**99.74**	**96.75**	**90.84**	**79.17**	**38.34**	26.83	−20.57
Experiment II: SEU Full 9-Class (mixed9)							
AF-iTransformer (Ours)	**99.89**	**95.83**	**91.62**	**78.58**	**38.18**	**31.36**	−21.31

**Table 5:** Ablation study on the UConn Gearbox dataset. Configurations tested with different tokenization strategies, frequency filter hidden dimensions, and individual module removals. $\Delta_{0dB}$ shows the accuracy drop from clean to 0 dB. Best values are highlighted in bold.

Configuration	Clean (%)	0 dB (%)	$\Delta_{0dB}$
AF-iTransformer (proposed)	**99.63**	**95.1**	−4.53
LinearStem, $H_{f}=64$			
w/o AdaptiveFreqFilter (NO_FREQ)	99.12	78.35	−20.77
w/o FiLM (NO_FILM)	99.08	86.52	−12.56
w/o Residual Attention (NO_RESATTN)	98.93	89.15	−9.78
w/o Aux Spectrum Head (NO_AUX)	99.21	90.87	−8.34
PatchStem, $H_{f}=32$ (FULL)	99.57	91.81	−7.76
LinearStem, $H_{f}=32$ (NO_PATCH)	99.75	91.58	−8.17
LINEAR (no extras)	98.58	85.46	−13.12

**Table 6:** Dual-branch mask analysis. Clean and 0 dB AWGN accuracy (%) for different α initializations (raw parameter values; the gate applies σ(α)). All configurations are learnable except the two “extreme” rows, which fix σ(α) at 1.0 (stats only) and 0.0 (global only). The best results per dataset are highlighted in bold.

Config	$\alpha_{\mathrm{init}}$	$\sigma {\left(\alpha \right)}_{\mathrm{final}}$	Clean (%)	0 dB (%)
UConn				
Global only	0.00	0.92	98.89	77.26
	0.25	0.94	98.52	84.04
Balanced (chosen)	0.50	0.99	**99.63**	**95.10**
	0.75	0.96	98.15	87.18
Stats only	1.00	0.96	98.52	85.95
Extreme stats only	—	1.00	98.15	81.64
Extreme global only	—	0.16	99.26	80.53
SEU				
Global only	0.00	0.00	98.44	64.81
	0.25	0.31	99.09	70.05
Balanced	0.50	0.52	94.14	61.53
	0.75	0.75	**99.74**	**78.58**
Stats only	1.00	1.00	98.83	74.44
Learned (from 0.25)	0.25	0.31	99.09	60.05

**Table 7:** Auxiliary loss weight sensitivity analysis. Test accuracy (%) at five SNR levels and best validation accuracy. The optimal λ=0.05 (in bold) achieves the best clean, 0 dB, −5 dB, and best validation accuracy. λ=0 corresponds to disabling the auxiliary head entirely.

λ	Clean	10 dB	5 dB	0 dB	−5 dB	−10 dB	Best Val
0.000	98.89	98.58	97.54	82.13	32.41	14.23	98.15
0.010	98.34	98.03	97.54	87.55	46.03	21.32	98.52
0.025	98.71	98.58	97.72	84.23	32.84	13.80	98.70
**0.050**	**99.63**	**99.47**	**99.12**	**95.10**	**56.56**	20.41	**99.99**
0.100	99.08	98.40	97.23	80.71	33.03	13.56	98.33
0.200	98.52	97.84	96.92	84.41	43.07	21.38	97.78

**Table 8:** Gradient monitoring on UConn with λ=0.05. Per-batch gradient norms and cosine similarity between the CE and auxiliary gradients on the shared encoder parameters (PatchStem + FiLM + encoder layers). Ten training-batch probes; mean ± std reported. The ratio $\lambda ∥\nabla L_{\mathrm{aux}}∥/∥\nabla L_{\mathrm{CE}}∥$ falls in the healthy regularizer range [0.05,0.5], and the cosine similarity is centered at zero, indicating the two gradients operate in complementary subspaces without conflict.

Metric	Mean ± Std	Min	Max	Healthy Range
$∥\nabla L_{\mathrm{CE}}∥$	0.0925 ± 0.0015	0.0896	0.0948	—
$∥\nabla L_{\mathrm{aux}}∥$ (raw)	0.2207 ± 0.0342	0.1593	0.2771	—
$\lambda ∥\nabla L_{\mathrm{aux}}∥$	0.0110 ± 0.0017	0.0080	0.0139	—
$\lambda ∥\nabla L_{\mathrm{aux}}∥/∥\nabla L_{\mathrm{CE}}∥$	0.1194 ± 0.0188	0.0848	0.1509	[0.05,0.5]
$\cos(\nabla L_{\mathrm{CE}},\nabla L_{\mathrm{aux}})$	+0.0008 ± 0.0109	−0.0139	+0.0214	>−0.2

**Table 9:** Convergence comparison on UConn: with vs. without the auxiliary spectrum head. Both configurations use the same seed, optimizer, scheduler, and augmentation. The auxiliary head reduces the epochs needed to reach 95% validation accuracy by 21% (19 vs. 23 epochs) and improves the best test accuracy by 0.37 pp, with negligible wall-time overhead.

Configuration	Epochs to 95% Val	Best Test (%)	Total Time (s)
With aux head (λ=0.05)	19	96.86	595.6
Without aux head (λ=0)	23	96.49	601.4
Speedup/gain	1.21×	+0.37 pp	1.01×

**Table 10:** FiLM statistics source comparison. Pre-filter statistics consistently outperform post-filter statistics on both datasets, with the gap largest on SEU (14.29-point drop at 0 dB). $\Delta_{0dB}={\mathrm{Acc}}_{0dB}-{\mathrm{Acc}}_{\mathrm{clean}}$.

Statistics Source	Clean (%)	0 dB (%)	$\Delta_{0dB}$
UConn			
Pre-filter (original)	99.63	95.10	−4.53
Post-filter (filtered)	96.85	89.45	−7.40
SEU			
Pre-filter (original)	99.74	79.14	−20.60
Post-filter (filtered)	89.43	64.85	−24.58

**Table 11:** Non-Gaussian noise robustness on the UConn Gearbox 9-class dataset. Test accuracy (%) under impulsive and harmonic noise at five SNR levels. All models were trained on clean data only; noise was applied exclusively at evaluation time. Best result per column within each block in **bold**.

Noise Type	Method	Clean	10 dB	5 dB	0 dB	−5 dB	−10 dB
Impulsive	1D-CNN	93.73	86.69	75.60	41.04	19.10	13.68
	ResNet-1D	97.98	96.00	90.76	61.98	20.27	11.83
	BiLSTM	98.71	97.23	95.87	82.56	42.02	**19.53**
	Vanilla iTransformer	99.46	99.08	**98.27**	**88.11**	43.93	18.18
	Transformer-1D	99.41	96.67	92.17	66.05	26.37	13.56
	AF-iTransformer (Ours)	**99.63**	**99.20**	97.60	82.56	**45.61**	18.42
Harmonic	1D-CNN	93.73	87.37	80.35	62.35	29.64	13.12
	ResNet-1D	97.98	94.09	78.99	40.73	21.44	14.36
	BiLSTM	98.71	96.80	95.56	83.55	40.36	18.42
	Vanilla iTransformer	99.46	98.64	98.09	91.99	55.70	21.07
	Transformer-1D	99.41	94.82	92.61	80.90	43.01	15.90
	AF-iTransformer (Ours)	**99.63**	**99.38**	**99.26**	**96.80**	**74.98**	**33.03**

**Table 12:** Non-Gaussian noise-robustness on the SEU planetary gearbox dataset. Experiment I: 5-class gear-only classification (gear5) under impulsive and harmonic noise. Experiment II: 9-class full-dataset evaluation under impulsive and harmonic noise. All models were trained on clean data only; noise was applied exclusively at evaluation time. Best result per column within each block in **bold**.

Noise Type	Method	Clean	10 dB	5 dB	0 dB	−5 dB	−10 dB
Experiment I: SEU Gearbox 5-Class							
Impulsive	1D-CNN	95.54	71.89	61.73	39.64	21.48	20.37
	ResNet-1D	98.47	73.89	64.56	41.61	23.04	21.64
	BiLSTM	99.52	76.29	66.88	44.56	**29.45**	**28.60**
	Vanilla iTransformer	99.65	77.23	68.37	47.49	27.26	26.63
	Transformer-1D	99.71	77.42	64.81	46.89	20.39	20.33
	AF-iTransformer (Ours)	**99.74**	**79.79**	**68.87**	**48.65**	27.67	26.09
Harmonic	1D-CNN	95.54	85.20	73.01	63.32	37.00	21.43
	ResNet-1D	98.47	88.93	79.95	66.06	40.91	22.80
	BiLSTM	99.52	89.45	80.20	71.05	**43.34**	27.52
	Vanilla iTransformer	99.65	91.34	85.46	**76.10**	39.76	**30.01**
	Transformer-1D	99.71	90.43	83.74	67.07	27.17	21.85
	AF-iTransformer (Ours)	**99.74**	**91.74**	**86.14**	75.37	38.04	29.15
Experiment II: SEU Full 9-Class							
AWGN	AF-iTransformer (Ours)	**99.89**	**95.83**	**91.62**	**78.58**	**38.18**	**31.36**
Impulsive	AF-iTransformer (Ours)	**99.89**	**89.35**	**77.09**	**72.87**	**16.95**	**16.71**
Harmonic	AF-iTransformer (Ours)	**99.89**	**95.62**	**89.75**	**80.39**	**39.23**	**22.47**

**Table 13:** Channel ablation on SEU dataset: clean accuracy (%) with different sensor subsets. The model degrades gracefully as channels are removed, and even single-sensor performance remains usable.

Channels	*S*	Clean (%)	0 dB (%)
All 8 channels	8	99.74	79.1
5 ch (vibration only)	5	98.57	77.45
3 ch (planetary XYZ)	3	97.92	75.95
1 ch (motor vibration)	1	98.05	75.15

**Table 14:** Per-window inference metrics across GPU and CPU devices. All timings use PyTorch with batch size = 1, auxiliary head disabled. Peak memory reflects maximum GPU allocation.

Dataset	Device	Params (K)	GFLOPs	Latency (ms)	Peak Mem (MB)
SEU	Quadro P620 (2 GB)	977	0.119	13.0	16.1
	GeForce MX550 (2 GB)	977	0.119	7.7	15.3
	RTX 4090 D (24 GB)	977	0.119	2.4	16.1
	i7-14700KF (CPU)	977	0.119	4.72	—
UConn	Quadro P620 (2 GB)	890	0.0034	7.3	12.8
	GeForce MX550 (2 GB)	890	0.0034	9.57	11.78
	RTX 4090 D (24 GB)	890	0.0034	2.4	12.8
	i7-14700KF (CPU)	890	0.0034	1.40	—

**Table 15:** Per-module parameter and latency breakdown of the proposed AF-iTransformer Param percentages are relative to training (including auxiliary head); latency percentages are relative to total GPU inference time on Quadro P620 (2 GB). Aux head is excluded at inference time. Encoder includes stem, FiLM, four attention layers, and a classifier.

	SEU			UConn		
Module	Params (K)	%P	Lat (ms)	Params (K)	%P	Lat (ms)
Adaptive Frequency Filter	141	13.3%	1.66	36	3.8%	1.33
FFT (time → freq.)	–	–	0.12	–	–	0.08
Mask MLP (Stats-cond.)	137	13.0%	1.40	36	3.8%	1.11
Global mask (learnable)	4	0.4%	–	0.5	<0.1%	–
iFFT (freq. → time)	–	–	0.14	–	–	0.14
Encoder Block	836	78.9%	8.00	854	90.9%	7.06
Stem (Patch/Linear)	11	1.1%	–	31	3.3%	–
FiLM conditioning	22	2.1%	–	22	2.3%	–
Residual Attn (×4)	793	74.9%	–	793	84.4%	–
Classifier head	9	0.8%	–	9	0.9%	–
Aux Spectrum Head (training)	83	7.8%	–	50	5.3%	–
Total (training)	1059	100%	–	940	100%	–
Total (inference)	977	92.2%	13.0	890	94.7%	7.3

## Data Availability

This paper uses the publicly available UConn dataset, which can be accessed through the following link: https://figshare.com/articles/dataset/Gear_Fault_Data/6127874 (accessed on 1 March 2026). The SEU dataset is publicly available at https://github.com/cathysiyu/Mechanical-datasets (accessed on 1 March 2026).
